# Raman Techniques: Fundamentals and Frontiers

**DOI:** 10.1186/s11671-019-3039-2

**Published:** 2019-07-12

**Authors:** Robin R. Jones, David C. Hooper, Liwu Zhang, Daniel Wolverson, Ventsislav K. Valev

**Affiliations:** 1 Turbomachinery Research Centre, University of Bath, Bath, BA2 7AY UK; 2 Centre for Photonics and Photonic Materials, University of Bath, Bath, BA2 7AY UK; 3 Centre for Nanoscience and Nanotechnology, University of Bath, Bath, BA2 7AY UK; 4 Department of Environmental Science and Engineering, Fudan University, Shanghai, 200433 China

**Keywords:** Raman spectroscopy, Hyperspectral microscopy, Spontaneous Raman scattering, Stimulated Raman scattering, Coherent anti-Stokes Raman scattering, Surface-enhanced Raman scattering, Tip-enhanced Raman scattering

## Abstract

Driven by applications in chemical sensing, biological imaging and material characterisation, Raman spectroscopies are attracting growing interest from a variety of scientific disciplines. The Raman effect originates from the inelastic scattering of light, and it can directly probe vibration/rotational-vibration states in molecules and materials. Despite numerous advantages over infrared spectroscopy, spontaneous Raman scattering is very weak, and consequently, a variety of enhanced Raman spectroscopic techniques have emerged. These techniques include stimulated Raman scattering and coherent anti-Stokes Raman scattering, as well as surface- and tip-enhanced Raman scattering spectroscopies. The present review provides the reader with an understanding of the fundamental physics that govern the Raman effect and its advantages, limitations and applications. The review also highlights the key experimental considerations for implementing the main experimental Raman spectroscopic techniques. The relevant data analysis methods and some of the most recent advances related to the Raman effect are finally presented. This review constitutes a practical introduction to the science of Raman spectroscopy; it also highlights recent and promising directions of future research developments.

## Introduction

### Raman Spectroscopy

There are numerous forms of light-matter interaction: fluorescence and phosphorescence are examples of absorption and subsequent emission of light by matter. Elastic scattering of light, such as Rayleigh scattering by atoms, molecules or phonons, and Mie/Tyndall scattering by dust particles are examples where the wavelength of the light is unchanged. Inelastic scattering such as Brillouin scattering by acoustic waves in crystals, Compton scattering by charged particles and Raman scattering by molecules or phonons are examples where the wavelength of the light does change [1]. Raman scattering of light by molecules was first predicted using classical quantum theory by Smekal in 1923 [2] and experimentally observed by Raman and Krishnan in 1928 [3, 4].

There are now more than 25 different types of known Raman spectroscopy techniques, such as spontaneous Raman, hyper-Raman scattering, Fourier transform Raman scattering [5], Raman-induced Kerr effect spectroscopy [6] and stimulated/coherent Raman scattering [1]. This review considers spontaneous and stimulated Raman scattering, coherent anti-Stokes Raman scattering (CARS), surface-enhanced Raman scattering (SERS) and tip-enhanced Raman scattering (TERS).

Fifty years after its first observation, Raman spectroscopy started to become a prominent analysis technique among other optical metrology techniques, such as those involving absorption of infrared light; particularly when water and other useful polar solvents were present, because these media typically strongly absorb light in the infrared region. For example, in 1974, Fleischmann et al. [7] used Raman spectroscopy to distinguish two types of adsorbed pyridine (a basic cyclic heterodyne compound molecule) on the surface of a silver electrode to mitigate absorption effects. This experiment was incidentally the first serendipitous observation of SERS.

Raman spectroscopy is now an eminent technique for the characterisation of 2D materials (e.g*.* graphene [8–10] and transition metal dichalcogenides [11–13]) and phonon modes in crystals [14–16]. Properties such as number of monolayers [9, 12, 17, 18], inter-layer breathing and shear modes [19], in-plane anisotropy [20], doping [21–23], disorder [10, 24–26], thermal conductivity [11], strain [27] and phonon modes [14, 16, 28] can be extracted using Raman spectroscopy.

The biological and medical fields of research are greatly impacted by the development of Raman spectroscopy as it is a label-free (does not require fluorescent marker molecules [29, 30]) chemically selective hyperspectral imaging technique [31]. For instance, studying the transdermal delivery of drugs into skin often ordains ex vivo and invasive analysis techniques. Ex vivo transdermal delivery studies are unfavourable because skin regeneration stops, the immune response ceases, and metabolic activity is usually lost. Hence, the performance of transdermal drug delivery ex vivo is not an accurate reflection of the in vivo situation [32]. However, non-invasive in vivo measurements can be performed using Raman spectroscopy to gain detailed information about the molecular composition and concentration gradients in the skin [33]. In many biological processes, living microorganisms such as bacteria act as biocatalysts. Raman spectroscopy can probe inhomogeneity in the properties and physiological status of individual cells in biocatalytic processes [34]. Raman spectroscopy has also been used to identify and differentiate benign and malignant breast cancer lesions by probing their unique chemical compositions [35].

For biological samples, approximately 90% of the peaks are found in the ‘fingerprint’ spectral region, covering ($\Delta \overset{\sim }{\nu }$ ~ 500 cm^−1^ to ~ 1800 cm^−1^; $\Delta \overset{\sim }{\nu }$ is the wavenumber shift defined in the “Analysis methods” section), with the remaining found in the higher energy CH/OH stretching vibrational modes covering ($\Delta \overset{\sim }{\nu }$ ~ 2700 cm^−1^ to ~ 3300 cm^−1^) [36].

### Coherent Raman Spectroscopy

Coherent light-scattering events involving multiple incident photons simultaneously interacting with the scattering material was not observed until laser sources became available in the 1960s, despite predictions being made as early as the 1930s [37, 38]. The first laser-based Raman scattering experiment was demonstrated in 1961 [39]. Stimulated Raman scattering (SRS) and CARS have become prominent four-wave mixing techniques and are of interest in this review.

SRS is a coherent process providing much stronger signals relative to spontaneous Raman spectroscopy as well as the ability to time-resolve the vibrational motions. SRS is relevant to numerous areas of research such as plasma physics [40], atomic interferometry [41], supercontinuum generation [42], imaging of biomolecules in food products [43], imaging chemistry inside living cells [44], bulk media and nanoscale specimens [45]. The exchange of photon orbital angular momentum by SRS in plasma is gaining interest, particularly in the context of inertial fusion research [40, 46–48]. Supercontinuum generation is a complex nonlinear phenomenon that is characterized by the dramatic spectral broadening of intense light pulses passing through a nonlinear material [49]. Knight et al. [42] demonstrated flat ultrabroadband octave-spanning white-light supercontinuum generation by SRS and parametric four-wave mixing with 60-ps pump pulses of sub-kilowatt peak power in a photonic crystal fibre. Kasevich and Chu [41] demonstrated a matter-wave interferometer with laser-cooled sodium atoms using the mechanical effects of stimulated Raman transitions. SRS has even been used to observe time-resolved vibrational spectra of the primary isomerisation of retinal in the visual pigment rhodopsin [50].

Since its resurgence in 1999, CARS has become a prominent vibrational mode imaging tool in biological medicine [51, 52]. As anti-Stokes photons are blue shifted from the pump and Stokes frequencies, they are more easily detected in the presence of single-photon fluorescence [53]. CARS microscopy has been successfully applied to live-cell imaging [51, 54], skeletal stem cells [55], tracing toxic nanomaterials in biological tissues [56], volumetric imaging of human somatic cell division [57], flow cytometry [58, 59], detection of brain tumours [60] and tracking organelle transport in living cells [61]. Zirak et al. [62] has developed a CARS endoscope for in vivo imaging and demonstrated the instrument with murine adipose tissue and human nervus suralis samples. Evans et al. [63] have combined CARS with video rate microscopy to chemically image tissue in vivo. Potma and Xie [64] have directly visualised lipid phase segregation in single lipid bilayers with CARS. CARS can even be used as a high temporal and spatial resolution thermography technique and has found applications in electronic and opto-electronic device characterisation [65] and even turbomachinery [66].

Orientational order is a salient feature of many soft matter systems. Detail in structural molecular organisation is a prevailing goal in the field of biology, biomedicine, material sciences and molecular physics [67]. Polarisation-resolved optical microscopy is becoming a powerful tool to address molecular orientational distributions into the focal volume of a microscope [68]. In coherent nonlinear optics, polarised second harmonic generation [69–71], polarised third harmonic generation [72] and polarised four-wave mixing [73, 74] have already been used to recover orientational information on endogeneous proteins and lipids in biological tissues. In addition to the orientational information, coherent Raman scattering (CRS) processes are sensitive to molecular bond vibrations, allowing chemical specificity without the need for fluorescence labelling/dyes [75–77]. CARS microscopy can be used to image chemical and orientational order of liquid crystalline (commonly used in display technology) samples [78]. Polarisation-resolved hyperspectral SRS microscopy has also been demonstrated as a label-free biomolecular imaging technique with teeth [79]. In addition, polarised-CARS has been used to study the molecular order of lipids in myelin at sub-diffraction scales in mice [80].

### Enhanced Raman Spectroscopy

The sensitivity of Raman spectroscopy can be enhanced through various techniques such as resonance Raman spectroscopy [81], TERS [82, 83] or SERS [84]. SERS is particularly interesting since it allows an enhancement of several orders of magnitude of the Raman signal by modifying the surface upon which an analyte material is to be placed. The enhanced light-matter interaction in TERS and SERS is tuneable (to some extent) by modifying the surface nanostructure of metallic films on dielectric surfaces [85, 86]. The wavelength of charge density oscillations, known as plasmons, is dependent on these surface nanostructures and can enhance the light-matter interaction locally [87]. Plasmons are a prominent topic in physics and plasmonic devices such as filters [88], waveguides [88, 89], polarisers [90] and nanoscale light sources [91] have now been realised.

Fleischmann et al. [7] first observed SERS in 1974 when investigating pyridine on the rough surface of a silver electrode [92]. The sensitivity of SERS makes it well-suited to study electron transfer reactions, which lie at the heart of numerous fundamental processes: electro-catalysis, solar energy conversion, energy storage in batteries, and biological events such as photosynthesis [93]. SERS has also been identified as a valuable technique for the detection of explosives/chemical weapons [94], unmodified DNA [95], aerosol pollutants [96] and pathogens [97].

TERS is a technique that provides spectral information with a spatial resolution on the nanometre scale [98]. Since the first reports of TERS emerged in 2000 [99, 100], TERS has become a powerful technique for studying thin crystalline materials [101], carbon nanotubes[86, 102, 103], single strands of RNA/DNA [104, 105], redox reactions [106], mapping of individual molecules [83], semi-conductor nanostructures and microcavities [107].

In the following sections, the fundamental physics that underpins the spontaneous Raman effect, stimulated- and coherent Raman spectroscopy, SERS and TERS are detailed in the context of their applications. Experimental considerations are discussed, and examples of Raman spectroscopy instrumentation setups are presented. The conventions for presenting spectra are detailed and examples of analysis techniques are given for each of the applications of Raman spectroscopy. In the final sections, the recent advances that constitute the current frontiers of Raman spectroscopy are presented from various fields of research worldwide.

## Fundamental Principles

When light interacts with matter, the oscillatory electro-magnetic (EM) field of the light perturbs the charge distribution in the matter which can lead to the exchange of energy and momentum leaving the matter in a modified state. Examples include electronic excitations and molecular vibrations or rotational-vibrations (ro-vibrations) in liquids and gases, electronic excitations and optical phonons in solids, and electron-plasma oscillations in plasmas [108].

### Spontaneous Raman

When an incident photon interacts with a crystal lattice or molecule, it can be scattered either elastically or inelastically. Predominantly, light is elastically scattered (i.e*.* the energy of the scattered photon is equal to that of the incident photon). This type of scattering is often referred to as *Rayleigh scattering*. The inelastic scattering of light by matter (i.e*.* the energy of the scattered photon is not equal to that of the incident photon) is known as the *Raman effect* [1, 4, 6]. This inelastic process leaves the molecule in a modified (ro-)vibrational state. In the case of a crystal lattice, the energy transfer creates a quantum of vibration in the lattice known as a *phonon* (a quasi-particle). Raman scattering in crystals can also lead to paramagnetic ions, surface plasmons (which are discussed later in this review) and spin waves [15]. The shift in angular frequency of the scattered light can be described by the following equation:

1 $$ {\omega}_{\mathrm{scat}}={\omega}_{\mathrm{p}}\pm {\omega}_{\mathrm{osc}}, $$

where subscripts osc denotes the lattice or molecule vibration, p denotes the incident photon (often referred to as the *pump photon*) and scat denotes the scattered light [1]. The binary operator (±) is determined by energy conservation. When the energy of the scattered photon is lower than that of the incident photon (i.e*.* red shifted), the process is referred to as *Stokes Raman scattering*. Conversely, when the energy of the scattered photon is higher than that of the incident photon (i.e*.* blue shifted), the process is referred to as *anti-Stokes Raman scattering*. The Raman process must also conserve momentum, which is expressed in wave vector form as:

2 $$ {\overset{\rightharpoonup }{k}}_{\mathrm{scat}}={\overset{\rightharpoonup }{k}}_{\mathrm{p}}\pm \overset{\rightharpoonup }{q}, $$

where ${\overset{\rightharpoonup }{k}}_{\mathrm{scat}}$, ${\overset{\rightharpoonup }{k}}_{\mathrm{p}}$ and $\overset{\rightharpoonup }{q}$ are the wave vectors of the scattered light, the incident light and the phonon or molecular (ro-)vibration, respectively.

In molecules and crystals, the charge distribution has an equilibrium state to which it tends. An externally applied field can modify or perturb the charge distribution but only in accordance with the molecule or crystal’s ability to form dipoles which may be anisotropic. This anisotropic property of molecules and crystals is called the polarisability and dielectric susceptibility, respectively. The classical approach theorises that the existence of the Raman effect is associated with the modulation of the polarisability (for molecular (ro-)vibrations) or dielectric susceptibility (for crystal lattice vibrations) due to the oscillatory nature of their interatomic displacement [6, 109]. For crystal lattice vibrations, consider the polarisation vector of the material, $\overset{\rightharpoonup }{P}$. If the suffixes *j* and *k* represent the vector components in the *x*, *y* and *z* directions, the *j*^th^ component of $\overset{\rightharpoonup }{P}$ (to *first-order*) is related to the oscillatory electric field vector $\overset{\rightharpoonup }{E}$ associated with the light by [110]:

3 $$ {P}_j^{(1)}={\varepsilon}_0{\chi}_{jk}^{(1)}{E}_k, $$

where *ε*_0_ is the permittivity of free space, *χ*_*jk*_ is the dielectric susceptibility of the material (a rank two tensor) and the convention of summation over repeated indices is implied [109]; the superscript (1) signifies that this is the first-order contribution to the polarisation [1]. The polarisability tensor is a function of the nuclear coordinates which, by extension, means that it will also depend on the (ro-)vibrational frequency. Assuming the modulation is small, the dependence can be expressed in a Taylor series with respect to the coordinates of vibration as follows:

4 $$ {\chi}_{jk}^{(1)}\left({\overset{\rightharpoonup }{k}}_{\mathrm{p}},{\omega}_{\mathrm{p}}\right)\approx {\chi}_{jk}^{(1)}{\left({\overset{\rightharpoonup }{k}}_{\mathrm{p}},{\omega}_{\mathrm{p}}\right)}_{\overset{\rightharpoonup }{u}=0}+{u}_l{\left(\frac{\partial {\chi}_{jk}^{(1)}\left({\overset{\rightharpoonup }{k}}_{\mathrm{p}},{\omega}_{\mathrm{p}}\right)}{\partial {u}_l}\right)}_{\overset{\rightharpoonup }{u}=0}+{u}_l{u}_m{\left(\frac{\partial^2{\chi}_{jk}^{(1)}\left({\overset{\rightharpoonup }{k}}_{\mathrm{p}},{\omega}_{\mathrm{p}}\right)}{\partial {u}_l\partial {u}_m}\right)}_{\overset{\rightharpoonup }{u}=0}+\dots, $$

where $\overset{\rightharpoonup }{u}$ is the nuclear displacement vector, the indices *j*, *k*, *l* and *m* indicate different spatial coordinates with repeated indices in any of the terms implying the summation of the constituents of that index. If we write the electric field associated with the light as follows:

5 $$ \overset{\rightharpoonup }{E}\left(\overset{\rightharpoonup }{r},t\right)=\overset{\rightharpoonup }{E}\left({\overset{\rightharpoonup }{k}}_{\mathrm{p}},{\omega}_{\mathrm{p}}\right)\cos \left({\overset{\rightharpoonup }{k}}_{\mathrm{p}}\bullet \overset{\rightharpoonup }{r}-{\omega}_{\mathrm{p}}t\right), $$

and the nuclear displacement as follows:

6 $$ \overset{\rightharpoonup }{u}\left(\overset{\rightharpoonup }{r},t\right)=\overset{\rightharpoonup }{u}\left(\overset{\rightharpoonup }{q},{\omega}_{\mathrm{osc}}\right)\cos \left(\overset{\rightharpoonup }{q}\bullet \overset{\rightharpoonup }{r}-{\omega}_{\mathrm{osc}}t\right), $$

an explicit expression for time dependence of ${P}_j^{(1)}$ can be found by substitution of these two mathematical equations of the monochromatic light and displacement. The numerous resulting terms pertain to optical processes such as Rayleigh scattering, optical absorption and Raman scattering. The term which pertains to the first-order Raman scattering is derived from the second term on the right-hand side of Eq. 4 and yields:

7 $$ {P}_j\left(\overset{\rightharpoonup }{r},t,\overset{\rightharpoonup }{u}\right)=\frac{1}{2}{\varepsilon}_0{\left(\frac{\partial {\chi}_{jk}^{(1)}\left({\overset{\rightharpoonup }{k}}_{\mathrm{p}},{\omega}_{\mathrm{p}}\right)}{\partial {u}_l}\right)}_{\overset{\rightharpoonup }{u}=0}{u}_l\left(\overset{\rightharpoonup }{q},{\omega}_{\mathrm{osc}}\right){E}_k\left({\overset{\rightharpoonup }{k}}_{\mathrm{p}},{\omega}_{\mathrm{p}}\right)\times \left\{\cos \left[\left({\overset{\rightharpoonup }{k}}_{\mathrm{p}}+\overset{\rightharpoonup }{q}\right)\bullet \overset{\rightharpoonup }{r}-\left({\omega}_{\mathrm{p}}+{\omega}_{\mathrm{osc}}\right)t\right]\bullet +\cos \left[\left({\overset{\rightharpoonup }{k}}_{\mathrm{p}}-\overset{\rightharpoonup }{q}\right)\bullet \overset{\rightharpoonup }{r}-\left({\omega}_{\mathrm{p}}-{\omega}_{\mathrm{osc}}\right)t\right]\right\} $$

This term contains sum (anti-Stokes) and difference (Stokes) frequencies and demonstrates conservation of momentum as per Eqs. 1 and 2. This formulation follows the classical description from refs. [1, 109].

**Fig. 1:**
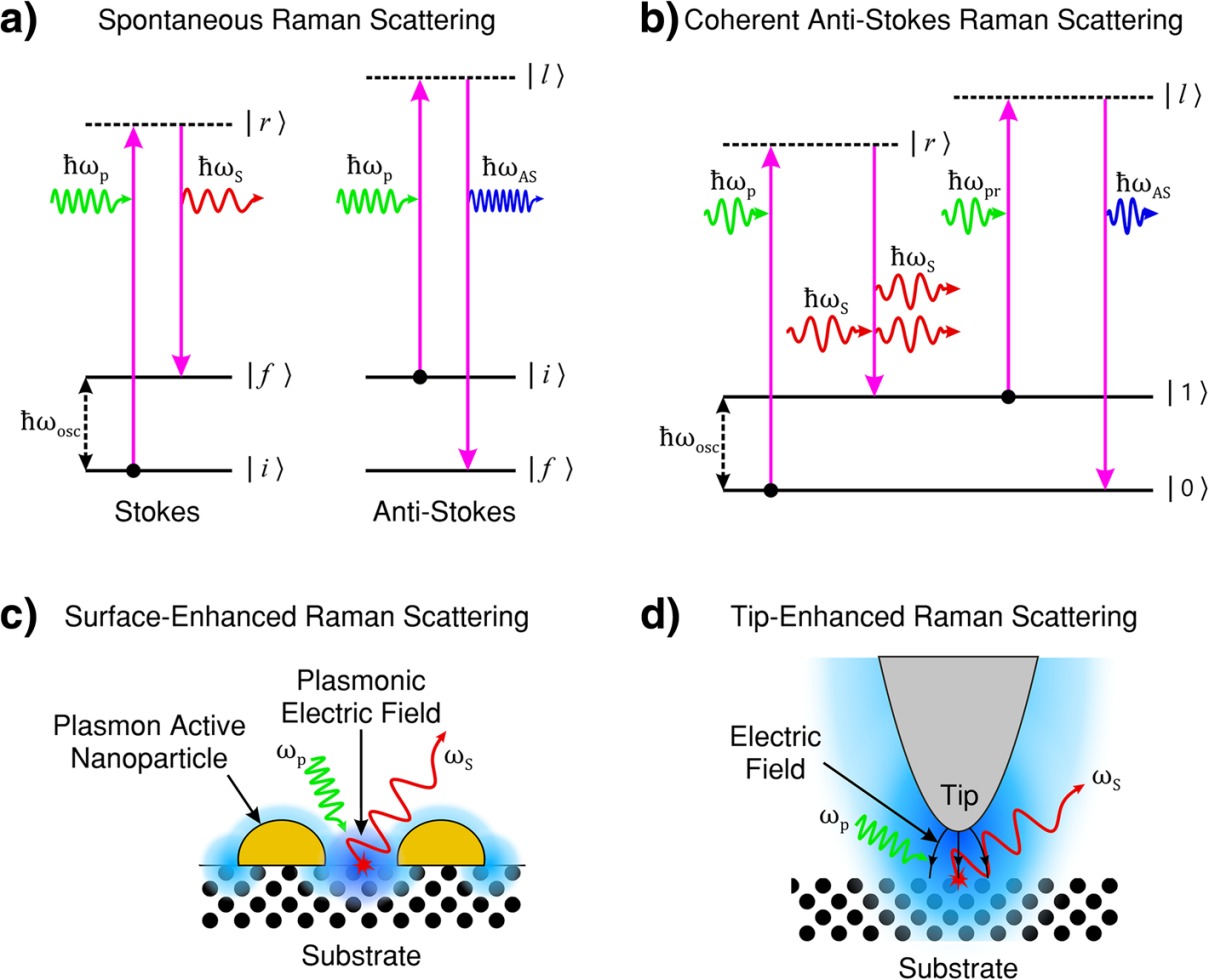
**a** Energy transfer process in Stokes (left) and anti-Stokes (right) Raman scattering, in both scattering processes, the lifetime of the excited state is probabilistic and spontaneous. In Stokes Raman scattering, the initial (ro-)vibrational energy *|i〉* of the scattering material is less than that of the final state *|f〉*, the scattered light has less energy than the pump light. In anti-Stokes scattering, the initial (ro-)vibrational energy *|i〉* of the scattering medium is greater than that of the final state *|f〉*, the scattered light has more energy than the pump light. **b** Coherent anti-Stokes Raman scattering (CARS). CARS is a four-wave mixing process of pump, Stokes, probe and anti-Stokes light in which the emission of anti-Stokes light is coherently induced through an intermediate (ro-)vibrational energy state population inversion. **c** Surface-enhanced Raman scattering (SERS). The incident pump light induces a surface plasmon resonance. The resultant enhancement of the oscillatory electro-magnetic (EM) field strength (shown in blue) on the surface intensifies the light-matter interaction and consequently increases the intensity of the Raman scattered light. **d** Tip-enhanced Raman scattering (TERS). The incident pump light induces a tip-surface plasmon resonance associated with the plasmonically active tip. The resultant enhancement of the oscillatory EM field strength (shown in blue) is localised to the vicinity of the tip apex. The lighting rod effect (illustrated by curved black arrows) intensifies the light-matter interaction in the tip region and provides high-resolution (beyond the diffraction limit of light) Raman imaging. **a**, **b** adapted from [1]. **c** adapted from [111]. **d** adapted from [112]

The quantum mechanical description of the Raman process states that the (ro-)vibrational energy of the molecules/phonons are discrete quanta. Figure 1a shows an energy level diagram illustrating the Raman processes with Stokes emission at *ω*_S_ and anti-Stokes emission at *ω*_AS_.

In Raman scattering, the intermediate states of the perturbation imposed by the incident pump photon (| *r* 〉 and | *l* 〉 in Fig. 1a, b) generally do not correspond to electronic states of the system and are said to be virtual energy states. These virtual intermediate states do not represent a well-defined energy state of the system. As the frequency of the pump photon approaches the energy of the electronic states, the strength of the Raman effect increases due to resonance effects and is termed pre-resonance Raman. If the intermediate state corresponds to a discrete electronic energy state, the interaction is described as resonance Raman scattering and the signal strength is expected to exceed that of virtual-intermediate-state Raman scattering by orders of magnitude. If the energy of the incident light is in the range of dissociative energy levels, the process is described as continuum resonance Raman scattering [1].

Raman scattering transitions between certain quantum states are forbidden. In materials with inversion symmetry (i.e*.* centrosymmetric crystal structure [113]), the initial and final states must have the same parity and are mutually exclusive with absorptive transitions (optically active transitions). In other words, transitions can be either Raman active or optically active. For linear molecules, the symmetric stretching modes of vibration or bending are Raman active and are optically inactive; those with anti-symmetric modes are Raman inactive and optically active (i.e*.* mutually exclusive) [1]. This rule is general and for nonlinear molecules, mutual exclusion is relaxed. In materials without inversion symmetry, (ro-)vibrational mode transition can be both Raman and optically active [1, 108].

The Stokes Raman signal for molecules is more intense than the anti-Stokes signal as the population of energy states is governed by thermal statistics [1, 108]. For bosonic systems, such as phonons in crystals, the probability of the scattering target occupying a given vibrational quantum energy state obeys Bose-Einstein statistics. Under nonresonant Raman scattering and thermal equilibrium, the ratio of the anti-Stokes and Stokes scattered intensity is given by [109]:

8 $$ \frac{I_{\mathrm{AS}}}{I_{\mathrm{S}}}={\left(\frac{\omega_{\mathrm{p}}+{\omega}_{\mathrm{osc}}}{\omega_{\mathrm{p}}-{\omega}_{\mathrm{osc}}}\right)}^4{e}^{\left(-\frac{\mathrm{\hslash}{\omega}_{\mathrm{osc}}}{kT}\right)} $$

where *I*_S_ and *I*_AS_ are the intensity of the Stokes and anti-Stokes light, respectively, *ℏ* is Planck’s constant divided by 2*π*, *k* is the Boltzmann constant and *T* is the temperature associated with the scattering species. This equation is sometimes used to measure the temperature via Raman spectroscopy [65]. This relation becomes inaccurate for resonance Raman scattering because the Stokes and anti-Stokes processes occur at different pump photon frequencies [109].

In the case of spontaneous Raman scattering, the Raman effect is very weak; typically, 1 in 10^8^ of the incident radiation undergoes spontaneous Raman scattering [6]. The transition from the virtual excited state to the final state can occur at any point in time and to any possible final state based on probability. Hence, spontaneous Raman scattering is an incoherent process. The output signal power is proportional to the input power, scattered in random directions and is dependent on the orientation of the polarisation. For example, in a system of gaseous molecules, the molecular orientation relative to the incident light is random and hence their polarisation wave vector will also be random. Furthermore, as the excited state has a finite lifetime, there is an associated uncertainty in the transition energy which leads to natural line broadening of the wavelength as per the Heisenberg uncertainty principle (*∆E∆t* ≥ *ℏ*/2) [1]. The scattered light, in general, has polarisation properties that differ from that of the incident radiation. Furthermore, the intensity and polarisation are dependent on the direction from which the light is measured [1]. The scattered spectrum exhibits peaks at all Raman active modes; the relative strength of the spectral peaks are determined by the scattering cross-section of each Raman mode [108]. Photons can undergo successive Rayleigh scattering events before Raman scattering occurs as Raman scattering is far less probable than Rayleigh scattering.

### Nonlinear Susceptibility

The polarisation described by Eq. 3 is in agreement with first-order (i.e*.* linear) optics and describes the single-photon scattering process (two-wave mixing process). In wave mixing processes with more than two waves, nonlinear optical polarisation must be considered due to the products of the mixed electric field components. Nonlinear optical polarisation can be described by [110] the following:

9 $$ {P}_j={\varepsilon}_0\left[{\chi}_{jk}^{(1)}{E}_k+{\chi}_{jk l}^{(2)}{E}_k{E}_l+{\chi}_{jk l m}^{(3)}{E}_k{E}_l{E}_m+\dots \right], $$

where *χ*^(2)^ is the second-order susceptibility (rank three tensor), *χ*^(3)^ is the third-order susceptibility (rank four tensor) and the sum over repeated subscript indices is again implied. Each of the terms in Eq. 9 can be written in shorthand by ${\overset{\rightharpoonup }{P}}^{(1)}$, ${\overset{\rightharpoonup }{P}}^{(2)}$, ${\overset{\rightharpoonup }{P}}^{(3)}$, etc. The physical processes that occur because of the second-order polarisation, ${\overset{\rightharpoonup }{P}}^{(2)}$, tend to be distinct from those arising from the third-order polarisation, ${\overset{\rightharpoonup }{P}}^{(3)}$. This polarisation can have electric dipole, quadrupolar, octupolar, (etc.) contributions. Under the electric dipole approximation, the second-order polarisation can only occur in crystals that are noncentrosymmetric (lack inversion symmetry). Hence, *χ*^(2)^ vanishes for media such as fluids (e.g*.* liquid/gas) and amorphous solids (e.g*.* glass). Third-order nonlinear optical interactions (i.e*.* those described by a *χ*^(3)^ susceptibility) can occur for both centrosymmetric and noncentrosymmetric systems [109, 110]. Electric quadrupolar, octupolar, (etc.) *χ*^(2)^ contributions do not disappear under inversion symmetry.

### Stimulated Raman Scattering

While spontaneous Raman scattering is an incoherent process, SRS is a coherent four-wave nonlinear optical mixing process. The modes of oscillation are in phase forming a coherent modulation of polarisation in the sample with susceptibility *χ*^(3)^(*ω*_S_; *ω*_p_ + *ω*_S_ − *ω*_p_) [110]. The scattered light is also coherent [45]. The SRS process is dependent on the spontaneous Raman cross-section, the spectral linewidth, the path length of the light-field-matter interaction, the input intensity and optical feedback (light generation) of Stokes frequency light [110].

**Fig. 2:**
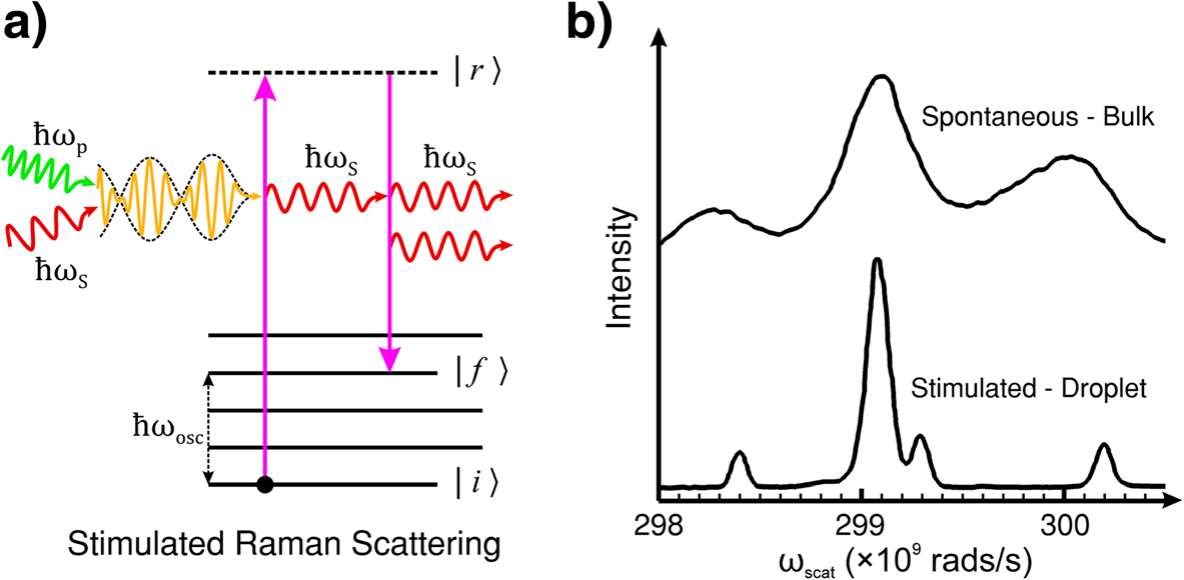
**a** Energy level diagram of stimulated Raman scattering (SRS). SRS is the induced emission of Stokes light by the coherent interaction of the pump and Stokes light with the material. Unlike spontaneous Raman scattering where the lifetime of the state *|r〉* and the energy of the final state *|f〉* are probabilistic, in SRS, the (ro-)vibration of the molecule or lattice is coherently driven by the difference frequency of the pump and Stokes light. **b** Comparison of spontaneous Raman scattering and SRS of bulk and droplet ethanol. The spontaneous measurements were performed in a cuvette (bulk ethanol). The SRS measurements were performed in a droplet of ethanol which acted as an optical resonator for the Stokes light. **b** reproduced with permission from the OSA [114]

When photons of frequency *ω*_p_ and *ω*_S_ simultaneously interact with a molecule or crystal lattice in the ground state, the system vibrates with an induced frequency: *ω*_osc_ = *ω*_p_ − *ω*_S_. Unlike spontaneous Raman scattering, the de-excitation (relaxation) time to and energy of the final state are determined by the stimulation effect. The interaction results in the transfer of energy from the pump photon to the molecule/lattice, and the molecule/crystal scatters a new photon with frequency and phase matching that of the incident light of frequency *ω*_S_. Figure 2a shows the process schematically.

It is common to employ an external radiation source tuned to the Stokes frequency in tandem with the pump laser beam to provoke this effect. This technique can lead to exponential gain in the Stokes signal, by transferring energy from the pump radiation, and rapid population of the final (ro-)vibrational state |*f* 〉 [115]. However, if the intensity of the incident light of frequency *ω*_p_ is sufficient, the generation of Stokes frequency photons within the material can self-promote SRS without the need for an external *ω*_S_ source. The intensity threshold of incident light in organic liquids, such as ethanol, for this kind of self-generated SRS typically requires an incident peak intensity of pump light > 10^9^ W/cm^2^ for an optical path length of a few centimetres. However, the SRS threshold can be significantly reduced by extending the length of the pump and Stokes field interaction with an optical resonator, such as internal reflection in a droplet of liquid. The example shown in Fig. 2b is the SRS spectrum taken with droplets of ethanol directly compared to the spontaneous Raman spectrum of bulk ethanol. The droplets act to confine the light by internal reflection which feeds back the Stokes light as a self-SRS inducing optical resonator [114].

### Coherent Anti-Stokes Raman Scattering

CARS is a third-order nonlinear four-wave optical mixing process. Figure 1b shows the energy level diagram for the process. A pump beam and probe beam of frequency *ω*_p_ and *ω*_pr_ are mixed with a third beam of frequency *ω*_S_ (Stokes frequency) and incident on the sample. The frequency difference (*ω*_*p*_ − *ω*_*S*_) needs to match the frequency associated with the Raman active (ro-) vibrational mode *ω*_osc_ = *ω*_p_ − *ω*_S_ [4, 53, 56, 116]. The frequency of the Stokes beam is usually adjusted/tuned to satisfy this criterion [117]. Next, a probe photon of frequency *ω*_pr_ provides a perturbation for the anti-Stokes scattering process to occur at frequency *ω*_AS_ = *ω*_p_ − *ω*_S_ + *ω*_pr_ [5]. A macroscopic third-order polarisation, *P*^(3)^, is induced due to the coherent superposition of the microscopic dipole oscillations [53]. Hence, CARS is governed by the third-order susceptibility of the form: *χ*^(3)^(*ω*_*AS*_; *ω*_*p*_ − *ω*_*S*_ + *ω*_*pr*_).

There are numerous treatments and approaches to formulating expressions for *χ*^(3)^. If one assumes that the excitation field is much weaker than the intramolecular forces, then a perturbative approach can be adopted [5, 110]. If this is not the case, non-perturbative treatments can be considered [118]. By considering the density matrix equation of the system and expressing the external field interaction as a perturbation in the Hamiltonian, the semi-classical nonlinear optics theory generates an expression for *χ*^(3)^ with 48 terms, each of which contribute to the third-order susceptibility [5]. A generalised expression for dominant terms in resonant CARS is given by the following [51, 119]:

10 $$ {\chi}^{(3)}=\frac{A_R}{\omega_{osc}-\left({\omega}_p-{\omega}_S\right)-i{\Gamma}_R}+{\chi}_{NR}^{(3)}, $$

where Γ_*R*_ is the half width at half maximum for the Raman line [51]; *A*_*R*_ is a constant representing the Raman scattering cross-section. The first term is the contribution due to CARS vibrational resonance as in Fig. 1b (*ω*_osc_ = *ω*_p_ − *ω*_S_). The second term is the nonresonant background signal and is independent of the Raman shift (*ω*_osc_ ≠ *ω*_p_ − *ω*_S_). The nonresonant background occurs because not all quantum pathways of the scattering process involve a resonance with a (ro-)vibrational state. This nonresonant contribution interferes with the resonant part of the signal. The nonresonant background causes distinctive distortions of CARS spectra in comparison with spontaneous Raman spectra and has prevented CARS from becoming a widespread technique [120].

The incident light beams of differing frequency move in and out of phase with each other in both time and space. Hence, the CARS signal reaches its first maximum when the field-sample interaction length scale is less than the coherence length scale to yield constructive interference [121, 122]. For *plane-wave* pump and Stokes beams, the intensity of the anti-Stokes signal is as follows [53, 123]:

11 $$ {I}_{\mathrm{AS}}\propto {\left|{\chi}^{(3)}\right|}^2{I}_{\mathrm{p}}{I}_{\mathrm{p}\mathrm{r}}{I}_{\mathrm{S}}{\left(\frac{\sin \left(\Delta  \overset{\rightharpoonup }{k}\bullet \frac{\overset{\rightharpoonup }{z}}{2}\right)}{\frac{\left|\Delta  \overset{\rightharpoonup }{k}\right|}{2}}\right)}^2, $$

where $\overset{\rightharpoonup }{z}$ is the sample thickness (vector normal to the lattice cell surface), $\overset{\rightharpoonup }{k}$ is the wavevector of light, $\Delta  \overset{\rightharpoonup }{k}={\overset{\rightharpoonup }{k}}_{\mathrm{p}}-{\overset{\rightharpoonup }{k}}_{\mathrm{S}}+{\overset{\rightharpoonup }{k}}_{\mathrm{p}\mathrm{r}}-{\overset{\rightharpoonup }{k}}_{\mathrm{AS}}$ is the wavevector mismatch (the velocity difference between the four waves) and *I*_i_ is the intensity of the wave denoted by the subscript. Phase matching is achieved when $\Delta  \overset{\rightharpoonup }{k}=0$ and the intensity of the anti-Stokes signal is maximised because the energy and momentum transfer processes correspond to allowed transitions. As the magnitude of *χ*^(3)^ is linearly proportional to the number oscillators involved in the process, the intensity of the anti-Stokes signal is quadratically proportional to the number/concentration of oscillators [53].

Researchers typically employ the pump beam to provide the second virtual excitation (i.e*.* the probe light shown in Fig. 1b; i.e ω_pr_ = *ω*_p_ and *ω*_AS_ = 2*ω*_p_ − *ω*_S_) [119]. The intensity of the CARS signal is therefore quadratically proportional to the intensity of the pump beam (see Eq. 11). The CARS signal is monodirectional due to the phase-matching condition [120]. However, high numerical aperture (NA) lenses or microscope objectives (confocal light) are commonly employed to satisfy the phase-matching condition without the need for complex mechanical systems to achieve phase-matched beam alignment [5, 117].

Raman resonances typically have coherence times of ~ 1 ps. Hence, the pump and Stokes beams are typically pulsed in picosecond timescales to obtain coherent excitation [124] and to inhibit multiphoton effects [53]. The CARS process takes place in the immediate vicinity of the beam’s focal spot. The signal produced is typically 10^6^ times that of spontaneous Raman scattering. CARS microscopy offers non-invasive characterisation and imaging of (ro-)vibrational spectra with high sensitivity and spectral resolution as well as three dimensional sectioning capabilities [116].

### Surface Plasmons and Polaritons

Surface plasmons can occur at the interface between a dielectric and conducting material, such as a metal or degenerate semi-conductor [88, 125]. They are the light-induced coherent oscillations of surface conduction electrons about their equilibrium position. The nanoscale volume of opposing charge that remains acts as a restoring force on the electrons. The result can be described with a damped simple harmonic oscillator model, in which the oscillations of the free-charge carriers have an associated resonance. Surface plasmons can be excited by EM radiation and *plasmonics* is the study of these light-matter interactions [126].

Plasmonic nanoparticles that are much smaller than the wavelength of the incident light can support non-propagating surface plasmons that oscillate with a frequency known as the local surface plasmon resonance (LSPR) [127, 128]. The wavelength of surface plasmons is much shorter than that of the associated propagating light for a given frequency [129]. The LSPR wavelength is dependent on the nanoparticle’s size, shape, material, external dielectric properties and inter-nanoparticle separation [85, 127, 128, 130–133].

Surface plasmons that propagate are referred to as surface plasmon polaritons (SPPs) [134–136]. They are essentially light waves that are trapped at the interface due to their interaction with the free electrons of the conducting material [88]. For a planar dielectric-conducting interface, polaritons propagate in 2-dimensional space along the surface interface for length scales of tens to hundreds of micrometres [126, 134–136]. They decay evanescently in the direction perpendicular to the surface interface with 1/e decay lengths of up to 200 nm [126, 137]. The field intensity in this evanescent decay region is amplified by orders of 10 to 100 relative to the incident radiation [136]. Hence, light-matter interactions with adsorbed molecules on the surface are also enhanced.

In the case of LSPR, the surface roughness or surface nanoparticles cause local concentrations of charge carriers which further amplify the evanescent EM field due to the lightning rod effect [138]. Even larger field-enhancements (up to 10^6^) can be observed in gap plasmons (in the gap between two neighbouring plasmonic nanoparticles; see Fig. 1c) [85, 111]. This enhanced near-field effect gives rise to the technique known as SERS and is discussed in the next section.

### Surface-Enhanced Raman Scattering

Raman is generally a very weak process; it is estimated that approximately one in every 10^8^ photons undergo Raman scattering spontaneously [6]. This inherent weakness poses a limitation on the intensity of the obtainable Raman signal. Various methods can be used to increase the Raman throughput of an experiment, such as increasing the incident laser power and using microscope objectives to tightly focus the laser beam into small areas. However, this can have negative consequences such as sample photobleaching [139]. Placing the analyte on a rough metal surface can provide orders of magnitude enhancement of the measured Raman signal, i.e*.* SERS.

Two mechanisms have been proposed to explain the increase in Raman signal provided by SERS. The first is via EM enhancements where local surface plasmons concentrate the local electric field near the surface of the metal in ‘hot spots’ located on the sharp edges of nanostructures or in regions of concentrated free-charge carriers due to the lighting rod effect [140]. Figure 1c, illustrates the SERS process. This process can increase Raman generation by a factor of 10^8^ to 10^11^ [86, 141–144]. The second method is chemical enhancement via charge transfer between the metal surface and the analyte, which enhances Raman scattering by a factor of approximately 10^2^ to 10^3^ [86, 145–147]. However, the charge transfer mechanism only applies to specific molecules, whereas the EM mechanism is applicable for all analytes [4, 92, 102, 148].

The ubiquity of EM enhancements has led to the development of numerous SERS substrates, which can be divided into two groups: metallic nanostructures fabricated on a solid substrate [85] and colloidal suspensions of plasmonic nanoparticles [96, 141]. The most common materials used to fabricate SERS substrates are gold and silver because of their good plasmonic response [149]. Gold also benefits from chemical stability as it is a noble metal. Other metals are also being investigated, such as aluminium for UV Raman spectroscopy [150, 151].

### Tip-Enhanced Raman Scattering

The diffraction limit of light restricts the focus spot size in standard optical techniques (such as Raman spectroscopy) to be at least half of the wavelength of the light according to Abbe’s criterion [152–154]. Light from the sample is composed of both propagating and non-propagating radiation. The non-propagating evanescent waves remain in the vicinity of their sources and do not participate in image formation in the far field. Instead, they extend laterally on the sample among the plasmon-active sites. Hence the spatial resolution is restricted by the size of the focal spot of the light. Even with a focal spot size of a half-wavelength (~ 250 nm for visible light), any objects that are much smaller than the half-wavelength would appear as a defuse shape.

TERS is a relatively new optical nanoimaging technique that combined Raman spectroscopy with scattering (or apertureless) scanning near-field optical microscopy. TERS offers spatial resolution far beyond the diffraction limit of the probing light. In the context of the a priori description, this is achieved by forcing the near-field evanescent light into the far-field image formation [86]. At the present date, the spatial resolution of TERS is typically reported to be 10–30 nm and is largely assumed to scale with the size of the tip’s apex [103, 155–157]. Incremental improvements to this resolution have been reported [104, 158]. Enhancement factors for TERS are significantly weaker than SERS due to the relative size of the probed signal volume. The enhancement factor (relative to spontaneous Raman scattering) is typically reported to be 10^3^ to 10^6^. As with SERS, two field enhancement mechanisms are thought to contribute to the Raman signal: EM and chemical enhancement [86].

TERS is implemented by positioning a plasmon-active (plasmonic) nanotip approximately 50 nm above the sample’s region of interest. The Raman probe light is focused onto the tip-surface cavity to induce LSPR within the tip’ apex and (in some circumstances) the sample surface. The surface plasmons may then enhance evanescent or near-field light with the incident probe light and/or the Raman scattered light. Hence, the LSPRs both confine and enhance the light field in the vicinity of the tip’s apex. The enhanced local EM field is most concentrated at the tip apex due to the lightning rod effect. This evanescent light at the tip apex can then excite or stimulate Raman, two-photon or second harmonic scattering from a nanoscale volume of the sample under the tip. A Raman image of the sample surface can be obtained by raster scanning the sample under the nanometric tip.

## Experimental Considerations

### Instrumentation

The nonresonant Raman effect is a very weak process. Hence, monochromatic, narrow-beam and high-intensity lasers are preferable to produce quality Raman spectra. The exploitation of microelectronics, such as stepper motor drives, photon counters, digital data acquisition and computational processing systems can further enhance the quality of spectra. As spontaneous Raman spectroscopy is naturally an incoherent process, continuous-wave laser sources are commonly used because pulsed lasers require higher peak powers for sufficient signal-to-noise ratio, which can photobleach/damage samples.

The choice of wavelength of the laser source depends on the required application. Lower visible wavelengths and UV cause strong photoluminescence in organic materials, which can mask the Raman peaks. Therefore, a longer visible or near-IR wavelength (500—830 nm) laser source is often suited for studying organic materials, because of the reduced photoluminescence. However, the Raman signal intensity is inversely proportional to the wavelength of the pump light. Hence, longer wavelengths of light require longer acquisition times [1, 6].

Raman spectroscopy is most often performed using laser sources at *λ* = 785 nm. This wavelength source is often selected as it balances the competing factors between Raman signal intensity, fluorescence, detector sensitivity and cost, and cost-effective/compact high-quality laser sources. However, visible lasers in the blue and green (e.g*. λ* = 532 nm) are becoming more common in Raman spectroscopy [159].

Raman scattering is measured in terms of the wavelength shift from the source wavelength. Ideally the illumination source for Raman measurements should be purely monochromatic, in other words, a single wavelength. However, all laser sources possess a spectrum of wavelengths known as a linewidth. The linewidth of a laser is usually measured in Hertz and is typically > 1 MHz for solid-state lasers used in Raman applications. A narrow linewidth is preferable for Raman spectroscopy because the measured shift in the Raman scattering process is limited by the laser’s linewidth.

Laser sources for Raman spectroscopy need to be stable in wavelength and power over extended periods of time and from use to use. Raman spectra are usually collected over long integration times and for many acquisitions. If the wavelength of the source drifts during a measurement, then the Raman peaks will drift as well, because Raman is measured as a shift relative to the pump light. Wavelength drift is also problematic from measurement to measurement as it causes peaks to shift, in turn making comparisons between measurements difficult. The output power stability of the source is important for similar reasons. If the laser power drifts from measurement to measurement, then quantitative comparisons cannot be made easily.

Spectral purity is another key criterion for Raman laser sources. The spectral purity of laser sources often requires side-mode suppression better than 60 dB. In many cases, side-mode suppression is sufficient if > 60 dB spectral purity is reached at ~ 1–2 nm from the laser wavelength peak. However longer wavelength (near-IR) Raman spectroscopy requires side-mode suppression ratios within a few hundreds of pm from the main peak. These criteria are discussed in the context of common Raman laser sources in the following paragraphs [159].

Most modern Raman systems use solid-state laser sources rather than gas lasers because of their spectral quality and stability. There are three main categories of continuous-wave solid-state laser sources used in Raman spectroscopy: Diode-pumped single-longitudinal mode (SLM) lasers; single-mode diode lasers (distributed feedback (DFB) or distributed Bragg reflection (DBR)); and volume Bragg-grating (VBG) frequency-stabilised diode lasers. These laser sources have varying optical characteristics.

Diode-pumped SLM lasers are readily available in compact form from the UV to the near-IR. Power levels of several Watts are achievable at 1064 nm in the near-IR. In the visible range, numerous lines in the blue-green-red region (457 to 660 nm) are available with output powers of ~ 100 mW. In the UV spectral range, power outputs of 10–50 mW at 355 nm are available. Hermite-Gaussian laser beam modes are described by their transverse electro-magnetic mode (TEM): TEM_m,n_, where m and n represent the Hermite-Gaussian mode index [46]. Diode-pumped SLM lasers provide excellent TEM_00_ mode beams, precise wavelengths with low drift, and a single-frequency linewidth > 1 MHz. The spectral purity of diode-pumped SLM lasers is typically > 60 dB in terms of their side-mode suppression ratio. Weak emissions that neighbour the laser’s main peak several nanometres in spectral shift can occur in diode-pumped SLM lasers. However, these neighbouring lines can be mitigated with dielectric band-pass filters. The wavelength of diode-pumped SLM lasers is typically stable to within 4 pm over a temperature change of 30 °C.

Single-mode diode lasers are compact and cost-effective pump illumination sources with single-frequency linewidth (> 1 MHz), single-TEM beam quality and output powers of up to ~ 100 mW. Wavelengths of *λ* = 785, 830, 980 and 1064 nm are most common in Raman spectroscopy. The side-mode suppression ratio is typically limited by sideband emission to ~ 50 dB at ~ 100 pm from the main peak.

VBG frequency-stabilised diode lasers use a narrow-linewidth VBG element with a diode-laser emitter to achieve narrow-line emission. These lasers are often used for applications requiring narrow-line emission at wavelengths that are not available for DFB or DBR laser sources. Frequency-locking multi-TEM diode lasers can be used to increase the output power of the narrow-linewidth emission. The stability of the output wavelength and linewidth requires careful thermomechanical control and high-precision alignment inside VBG frequency-stabilised diode lasers. Linewidths can range from single-frequency emission to ~ 10s of pm, depending on the wavelength and the output power. The side-mode suppression ratio is limited to ~ 50 dB, ~ 250 pm from the main peak emission. However, this can be improved using filters.

In confocal Raman imaging applications, it is necessary to use diffraction-limited TEM_00_ beams for optimum spatial resolution. However, this is relaxed for probe-based quantitative Raman analysis. In addition, confocal Raman setups require laser beam isolation as samples may generate optical feedback that is well aligned to the incident pump light. This counter-propagating feedback can induce power and noise instability and can even damage the laser source. Optical isolators are often integrated into the laser system itself because careful alignment must be achieved in the output after the isolator [4, 6, 159].

The spectrometer is a core component of any set-up used for measuring Raman spectra. The spectrometer should match the wavelength(s) of the laser source(s) used. The spectral range and resolution required will depend on the application. For example, the spectral range is determined by the position of the Raman peaks of interest (i.e*.* at large $\Delta \overset{\sim }{\nu }$ ~ 3000 cm^−1^or low $\Delta \overset{\sim }{\nu }$ ~ 1 cm^−1^). If the application requires closely spaced Raman peaks to be resolved, then spectral resolution is key. The spectral resolution of a spectrometer is largely determined by the slit width at the spectrometer entrance, the focal length of the spectrometer, the dispersion, the size of the grating (or prism) and the size and sensitivity/quality of the detector. There is a trade-off between the overall spectral range and resolution when considering the design of the experiment for a given application. In the case of weak Raman signals, optimising the signal-to-noise ratio is a priority.

Spectral filtering plays a vital role in the acquisition of Raman spectra. Firstly, the incident laser light must be spectrally pure, which is accomplished with a narrow-linewidth laser source as discussed previously. However, if the laser light is delivered to the sample by an optical fibre, then it is inevitable that Raman generation will occur in the fibre. Therefore, it is important to use a narrow band-pass filter to reject any Raman signal generated in delivering the laser to the sample. Narrow band-pass filters can provide transmission > 90 % at the laser wavelength while suppressing light to an optical density of OD > 5 at wavelengths differing by just 1% from the laser wavelength.

Importantly, light collected for detection requires filtering to block the laser wavelength. If the laser light is not filtered out, it can go on to generate Raman in the detection arm of the set-up and drown out the desired Raman signal when it reaches the spectrometer. The type of filter required depends on whether Stokes, anti-Stokes or both are to be measured. To only detect anti-Stokes Raman, a short-pass filter should be used as anti-Stokes Raman light has a higher energy and hence shorter wavelength than the laser source. To only detect Stokes Raman, a long-pass filter should be used as the Stokes Raman light has a lower energy and hence longer wavelength than the laser source. Long pass edge filters with edge-transition widths of < 3 nm and edge steepness < 40 cm^−1^ are available. To detect both Stokes and anti-Stokes Raman light, a notch filter centred on the laser wavelength should be used as it allows both shorter and longer wavelengths to be detected. Notch filters with OD > 6 at the laser line wavelength are available. Multi-notch filters are also available and can block multiple laser lines simultaneously. Holographic notch filters significantly outperform dielectric notch filters, providing excellent attenuation of the Rayleigh line while passing light as near as 50 cm^–1^ from the Rayleigh line. Acousto-optic modulators can also be used in conjunction with an excitation laser to select emissions with a desired wavelength (as a filter) [160] or as a time-gated illumination system in tapping mode atomic force microscopy (AFM)-based TERS [161].

The quantum efficiency of standard room-temperature silicon-based CCD devices for Raman signal detection degenerates above *λ* = 800 nm. For longer wavelengths, indium gallium arsenide array devices can be used, but these are less sensitive with higher noise levels and cost.

The visible to near-infrared wavelength range (*λ* = 500–830 m) is particularly suitable for inorganic materials (e.g. graphene, carbon nanotubes (CNTs) and fullerenes) and SERS. UV lasers are attractive for organic materials (e.g. pathogens, proteins, DNA, and RNA). For materials with strong fluorescence that require near-IR illumination, it is common to use a 1064-nm wavelength.

### Spontaneous and Coherent Raman Scattering Setups

Spontaneous Raman spectroscopy is most commonly used for modes with forbidden single-photon absorption or emission experiments [108]. SRS is sometimes used for wavelength shifting of coherent light, light amplification, pulse compression, phase conjugation and beam combining [108]. Unlike spontaneous Raman scattering, SRS is highly directional and offers enhanced signal strength and the ability to time-resolve the evolution and dephasing of coherent (ro-)vibrational motion [45].

**Fig. 3:**
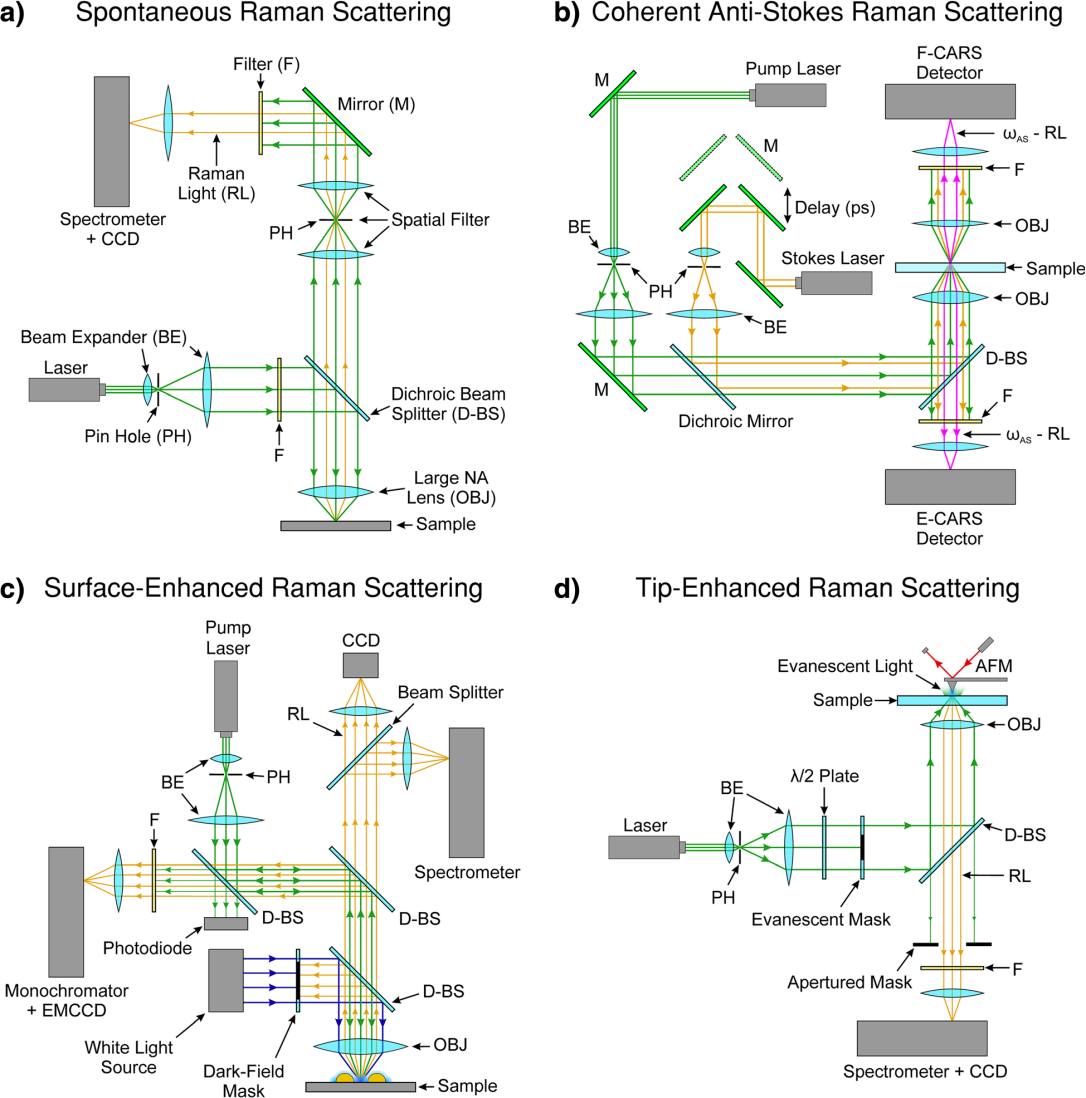
**a** Typical confocal Raman spectroscopy setup. The pump laser is spatially filtered through a pinhole. The back-scattered Raman light is spatially filtered and spectrally filtered through a notch filter. The Raman light is analysed by a spectrometer and a charge-coupled device (CCD). Hyperspectral images are obtained by raster scanning the sample. **b** Typical CARS setup. Two laser sources provide the pump and Stokes light and are synchronised through a picosecond path difference mirror setup. In this setup, the incident light is focused through an optically transmissive sample substrate. Both the forward scattered light (F-CARS) and epi-scattered light (E-CARS) are spectrally filtered by band-pass filters and are subsequently detected by two avalanche photodiodes. CARS images are obtained by raster scanning the sample. **c** Typical SERS setup. The pump laser is coupled into a dark-field microscope in which the Raman light is edge-filtered and detected through a monochromator and EMCCD. The white-light source and dark-field mask provides the means for dark-field spectroscopy. The dark-field spectra of each plasmonically active nanoparticle are recorded through a secondary spectrometer (top right in **c**). An imaging CCD camera is used to automatically find and centre each nanoparticle. **d** Typical TERS setup. The pump laser light is spatially filtered and passed through a half-wave plate. The evanescent mask ensures that only high numerical aperture (NA) pump light is incident on the sample such that total internal reflection occurs at the substrate-sample interface. This ensures that the tip apex is only illuminated by the evanescent light to achieve nanoconcentrated light in the vicinity of the tip. The reflected Raman light is filtered by an apertured mask (to remove any residual large NA pump light) and a notch filter. The Raman light is analysed by a spectrometer and a CCD. Hyperspectral images are obtained by raster scanning the sample. F, filter; M, mirror; RL, Raman light; CCD, charge-coupled device; PH, pinhole; BE, beam expander; D-BS, dichroic beam splitter; OBJ, Large numerical aperture (NA) lens; EMCCD, electron-multiplying charge-coupled device. **a** adapted from [162]. **b** adapted from [116]. **c** adapted from [85]. **d** adapted from [86]

Figure 3a shows a typical Raman setup based on a confocal geometry used by Wiedemeier et al. [162]. Confocal setups of this type are commonly used and employ an infinity-corrected objective lens (large numerical aperture (NA) lens) to focus the pump light. Wiedemeier et al. [162] used a diode-pumped solid-state laser as a monochromatic light source centred at 532 nm. Confocal mode is achieved by the use of a pinhole module in front of the spectrometer to spatially filter the light. The pinhole only passes light that originates from the focal plane to the detector. For detection of the Raman signal, a holographic-imaging spectrometer with an attached CCD camera is used. A holographic transmission grating with high light throughput served as a dispersive element, which enables large spectral ranges in a comparatively short time period to be acquired. Raster scanning of the sample in a confocal setup needs to be precise. Hence, a piezo actuated nano-positioner is used for positioning of the specimen.

Spontaneous anti-Stokes scattering is weaker than Stokes Raman scattering due to the relatively low probability of thermal excitation. Hence, anti-Stokes Raman spectroscopy is typically used with stimulated or coherent spectroscopy. CARS spectroscopy offers a 10^5^ increase in conversion efficiency, spectral and spatial discrimination against fluorescence and, most importantly, does not require a monochromator. Due to the required coherence of the process, high-peak power pulsed tuneable laser sources are employed. These peaks are readily available using picosecond or femtosecond light lasers, the choice of which is determined by the spectral resolution required and the timescale of interest [139].

Avoiding direct electronic excitations in the sample is an important consideration as photochemical damage (due to photobleaching) can occur in samples. Djaker et al. [139], for example, use near-infrared laser sources to mitigate photobleaching in their samples of polystyrene beads.

Figure 3b shows a typical CARS setup that measures both forward scattered light (F-CARS) and back- or epi-scattered light (E-CARS) [116, 139, 163]. The system has two synchronised picosecond pulse trains. The pump and Stokes beams are generated by two picosecond Ti:Sapphire lasers operating at 80 MHz and are tuneable from 700 to 1000 nm to cover the entire spectrum of molecular (ro-)vibrations in biological systems (up to $\Delta \overset{\sim }{\nu }$ ~ 3000 cm^−1^). The ps pulse duration is adjustable by a Gires-Tournois interferometer. The Ti:Sapphire lasers are pumped by a frequency-doubled CW Nd:Vanadate laser that provides monochromatic light at 532 nm. The two pulse trains were polarised with a pulse duration of 3 ps, corresponding to a spectral width of 1.76 cm^−1^. The pump and Stokes beams are synchronously pulse picked through two Bragg cells to reduce the repetition rate of the pulse trains to several hundred kilohertz, thus avoiding photodamage of the sample while still maintaining high-peak power for CARS generation. The pump and Stokes beams are temporally synchronised by a SynchroLock system, which electronically adjusts the time delay between the two pulse trains. A small part of the output of the lasers are launched in optical fibres coupled to photodiodes and connected to a SynchroLock controller, which measures the lasers frequency or phase difference between the master and the slave; the timing jitter was reported to be ~ 250 fs. The spectral resolution was estimated to be 2.5 cm^−1^, which is high enough to resolve Raman spectral features of biological samples. The use of a broadband Stokes wave enables the acquisition of a full CARS spectrum in only one measurement, with this configuration being known as multiplex or broadband CARS [164–167].

The two pulse trains are spatially filtered, collinearly combined and expanded through beam expanders. They are then sent into an inverted microscope and focused onto the sample by a water-immersion objective lens with a large NA. The E-CARS signal is collected by the same objective lens while the F-CARS signal is collected by a condenser lens with a lower NA. The E-CARS and F-CARS signals are filtered through a set of band-pass filters and detected by two avalanche photodiodes with a 200 μm × 200 μm active area. The CARS images are collected by raster scanning the sample, using an XYZ piezo flexure stage.

Several methods have been developed to suppress the nonresonant background associated with CARS. E-CARS is relatively insensitive to the nonresonant background of sample solvents [168]. Polarisation-sensitive CARS can differentiate the resonant and nonresonant signals by their polarisation [169]. However, these two techniques reduce the anti-Stokes signal strength [120]. Time-resolved CARS [170], temporal or spectral interferometry CARS [52, 171] and frequency-modulated CARS [172] can also suppress the nonresonant background. However, the setup in terms of both optics and electronics is challenging [120].

### SERS Specific Considerations

A variety of nanostructures, such as bowtie antennas [173], nano-rings [174], nanovoids [175], nanoparticle aggregates [87, 176, 177], nanoflower [178], nanorod arrays [97] and nanowells [179] can be used for SERS. Each nanostructure can have a number of plasmonic resonances, and matching the excitation laser to these wavelengths can greatly enhance the SERS intensity [141, 180, 181]. Matching the plasmonic resonance to the pump laser can be done either by tuning the laser wavelength or by tuning the LSPR of the nanostructures [85, 182–184].

The difficulty faced in producing SERS substrates is consistency in fabrication and repeatability in measurements due to the inhomogeneity and randomness of SERS active hot spots [85, 185, 186]. For SERS substrates produced by top-down methods, such as electron beam lithography [187], the main challenge is scaling the fabrication. Conventional top-down methods limit the active area of the SERS substrate and are not conducive to large-area manufacturing. Bottom-up fabrication methods have their own set of problems. Even though bottom-up approaches allow wafer scale fabrication, consistency across the wafer is usually lacking [188]. This inconsistency hinders the repeatability of measurements, which is problematic for quantitative analysis. Colloidal SERS schemes suffer from complications introduced by stabilising agents at the surface of the nanoparticles, which help to keep nanoparticles in suspension. These stabilising agents can either impede or augment the measured Raman signal [189]. The chemical synthesis for nanoparticle colloids also requires precise optimisation. The poor reproducibility of nanoparticle colloidal synthesis hampers batch-to-batch consistency.

Often, only very few sites exhibit the highest SERS enhancement and the variability in size and shape can alter the plasmonic properties from the desired LSPR [85]. Figure 3c, shows a setup which combines SERS with dark-field spectroscopy. The dark-field spectrometer analyses the light scattered from the nanostructures (illuminated by the white-light source) to select nanostructures with the desired plasmonic properties.

### TERS Specific Considerations

Scanning probe microscopy (SPM) techniques, such as atomic force microscopy (AFM), scanning tunnelling microscopy (STM) or shear force microscopy (SFM), are usually the tools of choice for TERS [86]. TERS has the ability to simultaneously measure topography by the conventional SPM mode of the system and obtain corresponding spectral information from a sample with nanometric spatial resolution and high sensitivity [86]. Certain SPM techniques ordain probe modifications for the plasmonically induced nanoscale evanescent light to activate/enhance the Raman signal. The tips can either be made of a metal or coated with a thin layer of metal to modify them for TERS. When the apex of a metallic or a metal-coated nanotip is illuminated with focused light at the LSPR wavelength, local surface plasmons around the tip apex are excited, and evanescent light is produced at the tip apex. This evanescent light can generate Raman scattering from a sample placed right under the tip apex. The process of Raman scattering takes place in the near-field and the spectral signal is scattered and converted back to the far-field by the tip apex, which is then collected by the usual optics and spectrometer in the far-field. Figure 3d shows such a TERS setup with a modified AFM. The setup consists of largely similar equipment shown in Fig. 3a (discussed in an earlier section). An inverted microscope illuminates the sample from underneath and the tip is placed at the top surface of the sample. The Raman back-scattered signal is then directed to the spectrometer. An evanescent mask blocks the central part of the laser beam inhibiting the low NA component of the incident light, so that only the high-NA component of the incident light reaches the sample so that total internal reflection occurs. This limits the transmitted light that falls onto the tip and, hence, only the evanescent light participates in the Raman scattering signal. Suppressing the participation of transmitted far-field light reduces the unfavourable background signal.

Polarisation-dependent TERS can be performed with light polarisation parallel to the tip apex in addition to the in-plane linear and radial polarisations. Polarisation dependent TERS is enabled by the large incidence angle from the high-NA objective lens and the use of devices that modify the polarisation state of the light such as a *λ*/2 waveplate [190]. The Raman scattered light is then collected in the low NA region through an apertured mask, which inhibits any residual laser light. As the tip apex approaches the sample within the focal spot, evanescent light is created at the tip’s apex [86]. Since the intensity distribution within laser focus is not uniform, it is very important to lock the relative position of laser focus to the tip [191, 192].

The strength and resolution of TERS depends on the ability of the tip to enhance and confine the light field at the tip’s apex, respectively. In STM systems, the tips are made of solid metal and the substrates need to be conductive in order to control the tunnelling current [193]. The STM tip resembles a long and smooth nanocone, with an apex diameter of ~ 20 nm. The length of the tip (~ tens of micrometres) makes them plasmonically unfavourable for visible light enhancement. However, the tunnelling gap between the tip and the sample can be tuned to the desired LSPR wavelength, creating a strong hotspot within the gap [143, 194, 195]. Some of the more advanced STM systems allow high-vacuum and low-temperature measurements [196]. As the substrate in STM needs to be conductive (often opaque in the visible wavelength range), the setup shown in Fig. 3d would not be suitable. Hence, a side illumination and side collection configuration is more common with STM-based TERS. To prevent the objective from mechanically interfering with the STM tip, a lens with a long working distance is required. It is therefore not trivial to tightly focus the incident light on the tip apex. A parabolic mirror can be used to mitigate mechanical interference and tightly focus the incident light to the tip apex as well as to collect the Raman signal [196, 197].

The spatial resolution in TERS is comparable to the size of the metallic nanostructure at the tip apex [86]. The gain in spatial resolution comes at a cost to overall signal enhancement (relative to SERS) due to the reduction of the Raman active volume.

**Fig. 4:**
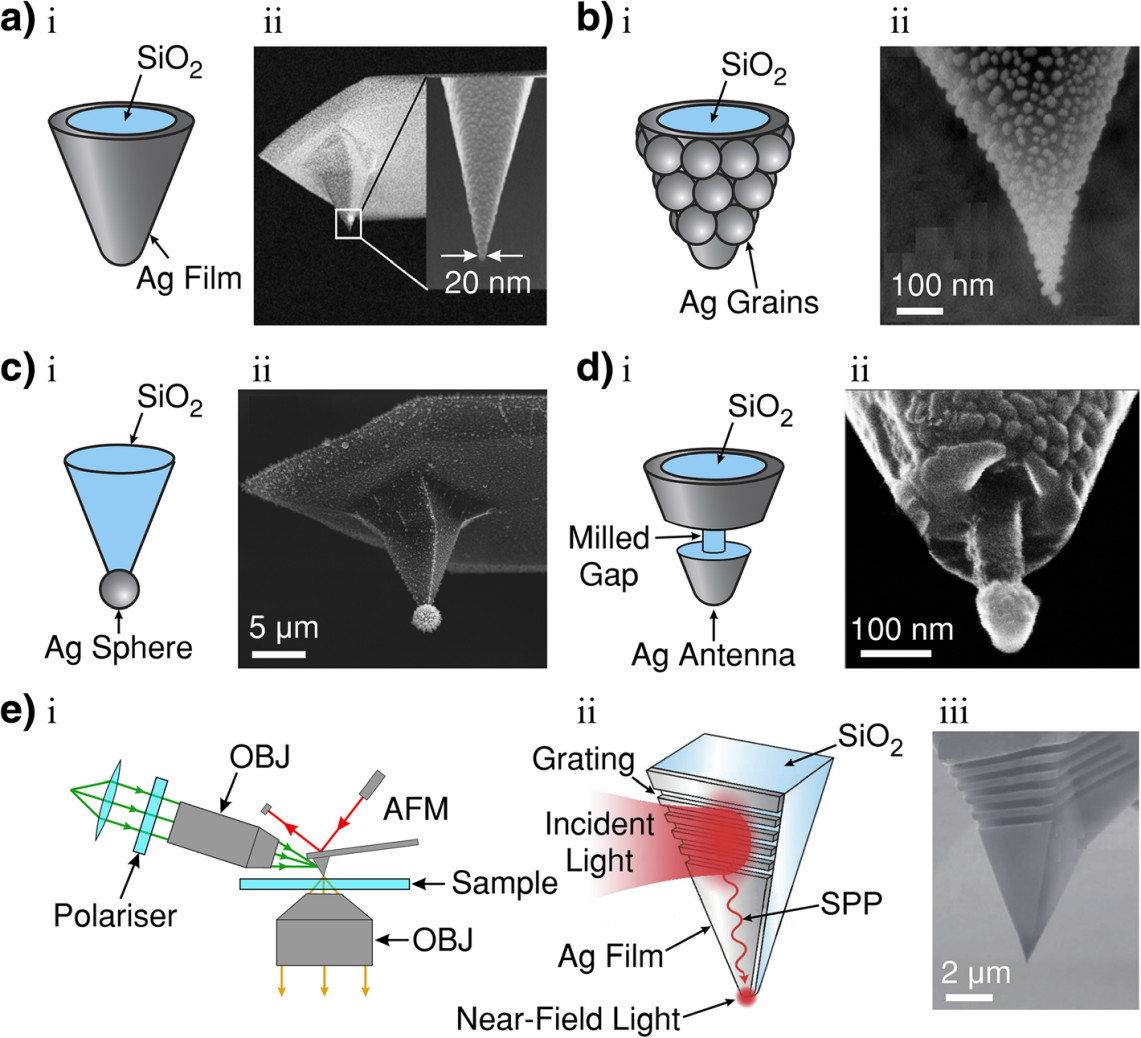
**a** i Smooth metallic (silver; Ag) film-coated dielectric (silicon-dioxide; SiO_2_) atomic force microscope (AFM) tip. **a** ii SEM image of a Ag-coated AFM tip. After Ag coating by thermal evaporation, a thin granular Ag layer is deposited onto the tip. **b** i, Rough Ag-nanoparticle-coated SiO_2_ AFM tip. **b** ii SEM image of rough Ag-grain-coated SiO_2_ AFM tip formed during the thermal evaporation process. **c** i Single Ag nanoparticle attached to the apex of a SiO_2_ AFM tip. **c** ii SEM image of an AFM tip after photoreduction to selectively fabricate an Ag nanoparticle at the tip apex. **d** i Ag-coated SiO_2_ AFM tip with a focused ion beam (FIB) milled gap. **d** ii SEM image of antenna fabricated by FIB milling of annular ring and subsequent Ag thermal evaporation from under the tip. The mushroom shape shadows the annular ring from Ag coating. **e** i Illustration of side illumination TERS for surface plasmon polariton (SPP) nanofocusing. OBJ, objective. **e** ii Schematic of the tip structure for SPP nanofocusing which is composed of a SiO_2_ pyramidal structure (AFM tip) and a Ag film on the surface. The incident light is coupled to the surface by the FIB-fabricated grating nanostructure. **e** iii SEM image of a Ag-coated SiO_2_ tip with a FIB-fabricated grating structure. **a** i, **b** i and ii, **c** i reproduced from Ref. [198] with permission from The Royal Society of Chemistry. (ref.). **a** ii reprinted with permission from [86]. **c** ii Reprinted from [199] with permission from IOP. **d** i Adapted from [200]. **d** ii Reprinted from [200] with permission from IOP. **e** i Adapted from [201]. **e** ii and iii reproduced from Ref. [201] with permission from The Royal Society of Chemistry

In AFM systems, the tips are usually semiconductor cantilevers, with an apex diameter of ~ 5 nm. Figure 4 shows five examples of AFM-based TERS tips that have been demonstrated in the literature. The semiconductor tips are usually coated with metal either by thermal evaporation under high-vacuum [202] or electroless metal plating (*mirror reaction*) [203] techniques. Figure 4a shows an example of a smooth AFM TERS tip. As the substrate does not need to be conductive, AFM-based TERS can be performed in either bottom-up transmissive illumination (as in Fig. 3d) or in side/top reflective illumination configurations; the transmissive configuration in Fig. 3d is more common.

The surface of AFM tips becomes nanostructured during the coating process resembling aggregated nanoparticles on the semiconducting tip (Fig. 4b) [198]. These nanostructures are suitable for the resonant excitation of LSPR and SPPs. The smooth tip shown in Fig. 4a has been fabricated by subsequently depositing a thin granular layer of additional metal. Other researchers have tested AFM tips with a metallic nanoparticle attached to the tip apex (Fig. 4c) [198], or a segregation in the tips coating to form an antenna (Fig. 4d) by focused ion beam lithography [115]. Tips can also be created by electrochemical deposition [204].

For transparent dielectric substrates, a thin metal film (thin enough to be transparent) can be coated onto the substrate to further enhance the field in the tip-sample gap [205]. It is also possible to perform TERS in liquids with AFM-based systems, which is favourable for biological specimens which require liquid environments to function [206]. Performing TERS in liquid with STM systems is much more difficult [86, 106]. SFM-based TERS is also an attractive technique and maintains many of the properties of AFM-based TERS with the exception of the tip material which resembles similar TERS properties of STM-based TERS [86, 156, 207, 208].

Some TERS setups have demonstrated vastly improved signal-to-noise ratio in TERS by SPP *nanofocusing* [201, 209, 210]. This technique focuses the laser onto a plasmon-coupling nanostructure (in the form of a grating) on the upper area of the tip, usually at a distance of ~ 10 μm from the tip apex. Figure 4e shows a typical nanofocused SPP-based TERS setup (i), the process of SPP nanofocusing by coupling the incident light to a focused ion beam-fabricated grating (ii), and an example SEM image of a SPP-nanofocusing tip (iii). The excited plasmons then propagate toward the tip apex through the process of adiabatic compression and create a confined EM field at the tip apex [209].

### Tuning the Plasmon Resonance

The size, shape, composition of the nanostructures and inter-nanostructure spacing all affect the wavelength of the surface plasmon resonance [85, 86]. Metals are most often used as the conducting medium for surface plasmons; however, semiconductors also possess plasmonic characteristics [125]. Gold shows strong enhancement factors in the red spectral region [111, 177, 211, 212], silver in the blue-green spectral region [132, 213] and aluminium in the UV and deep UV spectral regions [150, 151, 175]. The blue-green spectral region is the most commonly used Raman spectroscopy range. However, silver is prone to oxidation which degrades the plasmonic characteristics within a few hours of exposure to atmosphere. For this reason, silver is often mixed with other metals, such as titanium [214].

The range of plasmon resonance can be tuned by the thickness and choice of coating metal, e.g*.* tungsten, gold, silver or aluminium. In TERS, the grain size of the metal coating corrugations (Fig. 4b) is roughly comparable to the wavelength of the LSPR/SSP. Unlike STM tips, it is possible to control the LSPR/SPP wavelength by adjusting the size of the nanoparticles. The surface plasmon resonance wavelength is also dependent on the refractive index of the dielectric material. In AFM-based TERS, for example, the silicon cantilever tip can be heated to ~ 1000 °C in the presence of water vapour to oxidise the silicon into silicon dioxide [215]. As SiO_2_ has a lower refractive index than Si, the surface plasmon resonance is blue shifted [86].

The size and shape of the metal-coated AFM tip apex can also be modified to tune the LSPR [199, 200]. Fabricating a single metallic nanoparticle attached to the tip’s apex (Fig. 4c) or segregated antenna-shaped tip (Fig. 4d) has been demonstrated as a means to finely tune the surface plasmon resonance in AFM-based TERS [199, 200, 208, 216]. However, the most commonly used tips for AFM-based TERS are the tips that have disconnected metal nanoparticles evaporated on a semiconductor cantilever in the standard coating process (Fig. 4b) described a priori [198].

## Analysis Methods

### A Note on Units

By convention, Raman spectra are considered in terms of the wavenumber $\overset{\sim }{\nu }$ in units of cm^−1^. The conversion from angular frequency is as follows:

12 $$ \overset{\sim }{\nu }=\frac{\omega }{2\pi {c}_0}, $$

where *c*_0_ is the speed of light in vacuum and *ω* is the angular frequency. Raman spectra are usually plotted in terms of the wavenumber shift from the incident excitation radiation. This shift is defined as follows:

13 $$ \Delta \overset{\sim }{\nu }={\overset{\sim }{\nu}}_{\mathrm{p}}-{\overset{\sim }{\nu}}_{\mathrm{scat}}, $$

where ${\overset{\sim }{\nu}}_{\mathrm{p}}$ is the wavenumber of the pump beam with angular frequency *ω*_p_ and ${\overset{\sim }{\nu}}_{\mathrm{scat}}$ is the wavenumber of the scattered light accordingly. For Stokes Raman scattering, ${\overset{\sim }{\nu}}_{scat}={\overset{\sim }{\nu}}_p-{\overset{\sim }{\nu}}_{osc}$ (where ${\overset{\sim }{\nu}}_{osc}$ is the molecule or lattice vibration wavenumber) and $\Delta \overset{\sim }{\nu }$ is positive. By contrast, for anti-Stokes Raman scattering, ${\overset{\sim }{\nu}}_{scat}={\overset{\sim }{\nu}}_p+{\overset{\sim }{\nu}}_{osc}$ and $\Delta \overset{\sim }{\nu }$ is negative [1].

Raman spectra are (by standard) presented with the wavenumber shift linearly increasing from right to left on the horizontal axis. The vertical axis ordinate is linear and proportional to intensity. However, researchers also present Raman spectra with wavenumber shift denoted simply as wavenumber and/or increasing from left to right instead of right to left [1].

### Spontaneous Raman Spectra

**Fig. 5:**
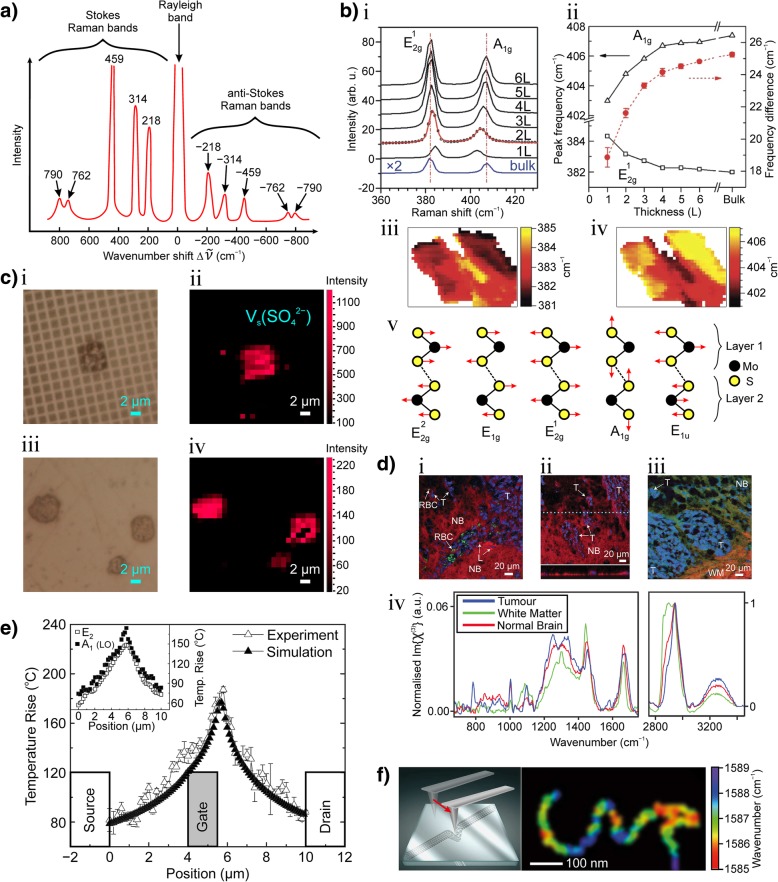
**a** Spontaneous Stokes and anti-Stokes Raman spectrum of carbon tetrachloride (liquid) excited by an argon ion laser, ${\overset{\sim }{\nu}}_{\mathrm{p}}=20487$ cm^−1^. The spectrum is presented according to recommendations of the International Union of Pure and Applied Chemistry. **b** i Raman spectra of thin multi-layer (nL) and bulk MoS2 films. The solid line for the 2 L spectrum is a double Voigt fit through data (circles for 2L, solid lines for the remainder). **b** ii Frequencies of ${\mathrm{E}}_{2\mathrm{g}}^1$ and A_1g_ Raman modes (left vertical axis) and their difference (right vertical axis) as a function of the number of layers. **b** iii, iv spatial maps (23 μm × 10 μm) of Raman frequency of ${\mathrm{E}}_{2\mathrm{g}}^1$ (iii) and A_1g_ (iv) from a sample of thin MoS2 films deposited on a SiO_2_/Si substrate. **b** v Atomic displacements of the four Raman-active modes and one infrared-active mode (E_1u_) in the unit cell of the bulk MoS2 crystal as viewed along the [1000] direction. **c** Microscopic image of nebulised ammonium sulphate aerosol particles on: i, Klarite; iii, silicon wafer. ii, iv Raman mapping image of sample (i) and (iii), respectively. **d** i Pseudo colour broadband CARS image of tumour and normal brain tissue, with nuclei highlighted in blue, lipid content in red and red blood cells in green. **d** ii Broadband CARS image and axial scan (below) with nuclei highlighted in blue and lipid content in red. **d** iii Broadband CARS image with nuclei highlighted in blue, lipid content in red and CH3 stretch–CH2 stretch in green. NB, normal brain; T, tumour cells; RBC, red blood cells; L, lipid bodies; WM, white matter. **d** iv Single-pixel spectra. **e** Raman thermography measurements across the active region of a high electron mobility transistor on SiC substrate with both E_2_ and A_1_ (LO) phonons considered to compensate for thermal stress. Device temperature rise determined using either E_2_ or A_1_ (LO) phonon mode alone (neglecting thermal stress) is shown in the top left insert. **f** (left) illustration of the manipulation of a straight isolated carbon nanotube (CNT) lying on a glass substrate by the sharp apex of an AFM tip. **f** (right) two-dimensional image of a CNT constructed by colour-coding the frequency position of the G+ vibrational mode in TERS spectra. The colour variation shows the strain distribution along the CNT at high-spatial resolution. **a** reproduced with permissions from [1]. **b** Adapted with permission from [217]. **c** Reprinted with permission from [96]. **d** Reprinted by permission from [167]. **e** Reprinted from [218]. **f** Reprinted by permission from [219].

Figure 5a shows the Rayleigh and the Raman spectrum of carbon tetrachloride (liquid) excited by an argon ion laser, ${\overset{\sim }{\nu}}_1$ ~ 20,487 cm^−1^ (487.99 nm). This spectrum is presented according to recommendations of the International Union of Pure and Applied Chemistry. It contains a strong band at ${\overset{\sim }{\nu}}_1$ ~ 20,487 cm^−1^ due to the Rayleigh scattering of the incident laser radiation and a number of weaker bands with wavenumbers, ${\overset{\sim }{\nu}}_1\pm {\overset{\sim }{\nu}}_{osc}$: ${\overset{\sim }{\nu}}_{osc}$ = 218, 314, 459, 762 and 790 cm^−1^. The Stokes Raman lines are shown on the left-hand side of the plot (Fig. 5a); the anti-Stokes Raman lines are shown on the right. The ${\overset{\sim }{\nu}}_{osc}$ values relate to the fundamental vibrations of the carbon tetrachloride molecule [1]. In the original work by Raman and Krishnan [220], the same spectrum was measured using mercury arc radiation (${\overset{\sim }{\nu}}_1$ = 22,938 cm^−1^, 435.83 nm). In this seminal work, the anti-Stokes bands at ${\overset{\sim }{\nu}}_1+762$ and ${\overset{\sim }{\nu}}_1+790$ cm^−1^ were not observed. Hence, after the invention of the laser, Rayleigh and Raman scattering experiments are preferably performed using monochromatically intense lasers.

### Layered Two-Dimensional Systems

Raman spectroscopy can be used to determine the layer thickness in two-dimensional materials with atomic level precision, using either the inter-layer or intra-layer vibrational modes [19]. Lee et al. [12] demonstrated the technique with two intra-layer Raman modes of molybdenum disulphide (MoS_2_). Figure 5b shows representative Raman spectra for single- and few-layer MoS_2_ samples. Among the four Raman-active modes of bulk 2H phase MoS_2_ crystal (shown in Fig. 5b v), Lee et al. [12] only observed the ${E}_{2g}^1$ and *A*_1*g*_ modes near $\Delta \overset{\sim }{\nu }$ = 400 cm^−1^. The authors surmised that the other modes were not observed either because of the selection rules for the scattering geometry (*E*_1*g*_) [217] or because of the limited rejection of the Rayleigh scattering radiation (${E}_{2g}^2$) [13]. The authors [12] report that single-layer MoS_2_ exhibits a strong in-plane vibrational mode at $\Delta \overset{\sim }{\nu }$ ~ 384 cm^−1^, corresponding to the ${E}_{2g}^1$ mode of the bulk 2H-MoS_2_ crystal. For all film thickness, the Raman spectra in Fig. 5b i show strong in-plane ${E}_{2g}^1$ and out-of-plane *A*_1*g*_vibration signals. As the sample thickness increases (Fig. 5b i and ii), the ${E}_{2g}^1$ mode red shifts and the *A*_1*g*_ mode blue shifts. For films of four of more layers, the ${E}_{2g}^1$ and *A*_1*g*_ modes converge on the bulk values. Spatial maps of a MoS_2_ film sample for the ${E}_{2g}^1$ mode is shown in Fig. 5b iii; that of the *A*_1*g*_ mode is shown in Fig. 5b iv. These maps demonstrate that the frequency of the two modes only slightly vary in regions of the sample with a given layer thickness. Hence, Raman spectra can provide a convenient and reliable means of determining the layer thickness in two-dimensional crystalline materials with atomic level precision.

### Enhanced Raman Scattering Through SERS

Ault et al. [221] were the first to use SERS to enhance the Raman scattering signal of previously undetectable secondary organic aerosol particles on Ag nanoparticle-coated quartz substrates. Fu et al. [96] demonstrated enhancement factors of 6 for the Raman spectra of ammonium sulphate (AS) at the Raman active mode ${\overset{\sim }{\nu}}_{\mathrm{s}}$(SO_4_^2−^) at 970 cm^−1^ with Klarite. Figure 5c shows a microscope image of a large AS particle on the surface of Klarite (Fig. 5c i), the corresponding Raman mapping image is in Fig. 5c ii. Figure 5c iii shows another sample of AS particle but on a silicon wafer. The corresponding Raman mapping image is shown in Fig. 5c iv. Aside from the three larger AS particles, small (sub-micron) AS particles are apparent in Fig. 5c iii. However, in the absence of SERS, these smaller particles are undetectable. On the other hand, the SERS Raman mapping image (Fig. 5c ii) shows a vastly enhanced signal intensity, as is evident from the scale bars, to the point where a number of small spots yield a signal at the ${\overset{\sim }{\nu}}_{\mathrm{s}}$(SO_4_^2−^) Raman mode. Such spots most likely correspond to small AS particles that are observable in Fig. 5c ii but are not apparent in Fig. 5c i.

### Insights into Cellular Structure with CARS

CARS microscopy is relevant to the chemical [64, 222–225], materials [78, 226, 227], biological and medical fields [29, 36, 61, 63, 167, 228] and can provide unparalleled insights into cellular structures [53]. Spontaneous Raman and infrared micro/spectroscopy can provide adequate chemical specificity and sensitivity to delineate a variety of neoplasms [229–237] but require long integration times and have a coarse spatial resolution, which may limit accurate tumour-boundary identification and early-stage tumour detection. However, coherent Raman imaging techniques have demonstrated high-speed, high-spatial-resolution imaging, but with contrast limited to single or few Raman peaks [36, 53, 167, 232]. Figure 5d presents images of orthotopic xenograft brain tumours from within a murine brain [167]. Figure 5d i shows a broadband CARS image with nuclei in blue ($\Delta \overset{\sim }{\nu }$ = 730 cm^−1^), lipid content in red ($\Delta \overset{\sim }{\nu }$ = 2850 cm^−1^) and red blood cells in green ($\Delta \overset{\sim }{\nu }$ = 1548 cm^−1^ + 1565 cm^−1^: C-C stretch from haemoglobin [238]). The large tumour mass and a projection of neoplastic cells within healthy tissue are clearly shown (Fig. 5d i). Figure 5 ii shows several small regions of main tumour mass migrating into the healthy brain matter. Figure 5 iii shows the boundary between normal brain tissue, white matter and tumour masses, which contrasts lipids in red ($\Delta \overset{\sim }{\nu }$ = 2850 cm^−1^); CH_3_ stretch-CH_2_ stretch ($\Delta \overset{\sim }{\nu }$ = 2944 − 2850 cm^−1^), a general contrast; and nuclei in blue ($\Delta \overset{\sim }{\nu }$ = 785 cm^−1^). The image shows the fibrous texture of the white matter and strands of myelination around cancer cell clusters. Figure 5d iv presents a set of single-pixel spectra from an intra-tumoural nucleus, the white matter and normal brain, respectively. The spectra indicate that lipids are most concentrated in the white matter and least in the tumour regions.

### Raman Thermography

Advances in electronic and opto-electronic semiconductor devices, such as high electron mobility transistors (HEMTs), have led to thermal management challenges [65]. Conventional thermal characterisation approaches such as infrared thermography are often no longer applicable for the accurate characterisation of high-power density devices due to limited spatial resolution which can result in the underestimation of the device peak temperature [239]. Batten et al. [218] have demonstrated temperature profiling in AlGaN/GaN HEMTs using Raman thermography by exploiting the E_2_ and A_1_ (LO) phonon modes. Both the E_2_ and A_1_ (LO) modes shift to lower frequency when operating the device. Figure 5e shows a comparison of the temperature rise in a AlGaN/GaN HEMT on a SiC substrate from Raman thermography and thermal simulations. The device was operated at a source-drain voltage of 40 V and a power density of 25 W/mm and had a thermal resistance of 8 °C/(W/mm).

### Measuring Strain on the Nanoscale Using TERS

TERS microscopy is an effective means of imaging nanostructures beyond the spatial resolution of the so-called light diffraction limit [152–154, 219]. Nanostructures such as DNA molecules [240], carbon nanotubes (CNTs) [241, 242], silicon devices [101, 243], dye molecules [244] and single molecules [83] can be imaged using TERS. The technique can even be used to measure the local molecular strain in nanostructured materials. For example, AFM can be used to manipulate CNTs with nanoscale precision to develop a local strain [245–248]. Figure 5f (left) illustrates the process of CNT manipulation using contact-mode AFM. Although local strain in CNTs has previously been studied using AFM and transmission electron microscopy [245], TERS microscopy is the only optical technique that can provide images of such local structural distribution of nanomaterials. When a straight CNT is deformed by manipulation, a local breakdown in symmetry is induced. This causes the selections rules of Raman scattering to become relaxed, allowing forbidden Raman modes to become visible in the vicinity of the local curvature [219]. The position of the characteristic G-mode Raman scattering line in graphene can be used to deduce local strain using TERS [249]. Figure 5f (right) shows a TERS image of a deformed CNT which has been constructed from the peak positions of the G^+^-mode [219]. The image has a spatial resolution better than 20 nm which is about 25 times finer than the diffraction limit of the excitation wavelength of light (488 nm). The colour variation (as indicated by the scale bar) corresponds to the local peak position of the G^+^-mode and represents the variation of strain along the CNT.

## Recent Results

### Stimulated Raman Scattering Microscopy

Unlike CARS, SRS microscopy does not contain a nonresonant background signal that degrades image contrast. However, SRS can be affected by cross-phase modulation (where light at one wavelength modulates the refractive index in the medium affecting another wavelength of light), transient-absorption (which is characteristic of femtosecond light pulses) and photo-thermal effects which can modify the vibrational energy levels and reduce hyperspectral image contrast [250–252]. SRS is quantified by the amount of energy transfer from the pump light to the Stokes light when the difference frequency between the pump and Stokes light matches a specific vibrational frequency, *ω*_osc_. In addition, the resulting signal from SRS is strongly sensitive to the incident polarisations when the orientation of the probed vibrating species is ordered. This polarisation dependence can be exploited to probe the orientational order of vibrational modes in samples. However, currently developed techniques are not able to perform large-field fast time scale dynamics instantaneously due to the requirement of point-wise scanning over the sample space. Conventional polarisation-resolved techniques take minutes because each point of the scanning area must be polarisation tuned sequentially [74, 80, 253, 254].

Multi-lamellar myelin plays a crucial role for efficient transmission of nerve impulses as an electrical insulator [255]. The lipids and proteins in myelin self-assemble into a highly ordered and stable structure to form a tightly packed membrane [256]. In neurological disorders, this compact structure is highly perturbed leading to dysfunctions of the central nervous system [257, 258]. As these biological processes are highly dynamic, researchers seek to observe the dynamics of molecular order with sufficient resolution and frame rate. Hofer et al. [259] have recently demonstrated fast-polar-SRS by exploiting high-speed amplitude- and polarisation modulation with an acousto-optic modulator (AOM) and electro-optical polarisation modulation, respectively, to read out the molecular order and orientation at a fast rate. They therefore obtain both amplitude and phase information. The authors report the ability to retrieve density maps of molecular bonds with the absolute value of molecular order. The linear polarisation direction of the pump beam is rapidly rotated while the Stokes polarisation is circularly polarised to avoid polarisation dependence from the Stokes beam. The polarisation is further modified by a quarter-wave plate. The polarisation modulation leads to an *α*-dependant response of the signal intensity given by the following:

14 $$ I\left(\alpha \right)\propto {a}_0+{S}_2\cos 2\left(\alpha -{\varphi}_2\right) $$

where *α* is the rotating pump polarisation direction in the sample plane, *a*_0_ is the total measured intensity, and *S*_2_and *φ*_2_ are the amplitude and phase of the second-order induced modulation [259].

**Fig. 6:**
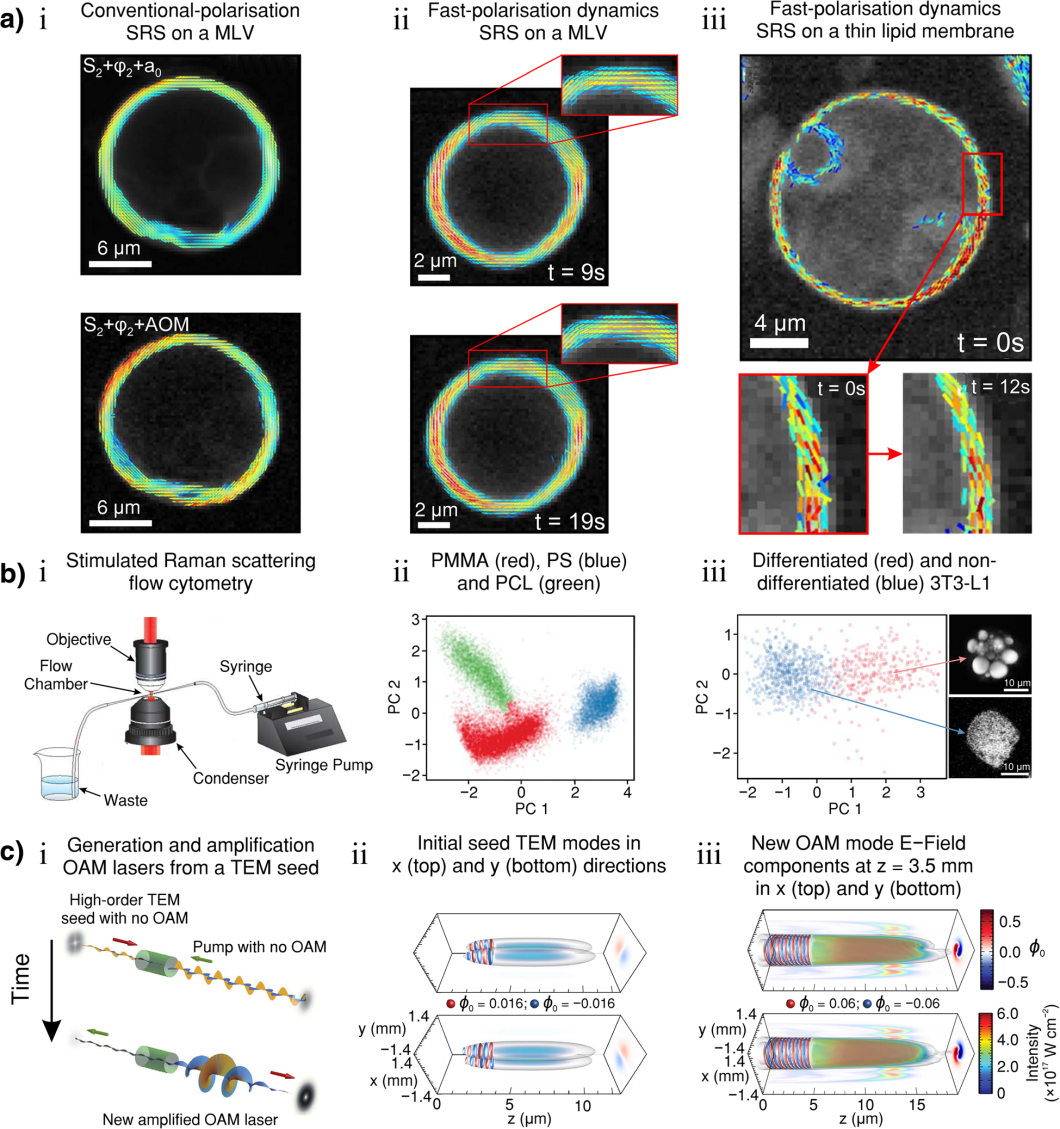
**a** Comparison of conventional and fast-polarisation SRS on multi-lamellar lipid vesicles (MLVs). **a** i–iii the composite images show absolute local molecular order (S_2_) and mean molecular orientation (*φ*_2_) values represented as coloured sticks. **a** i (top) Conventional polarisation SRS on a MLV using step angles of 5° (acquisition time 112 s); the total measured intensity (*a*_0_) is represented as a grey-scaled background. **a** i (bottom), fast-polarisation SRS on the same MLV (acquisition time 1 s); the acousto-optic modulation (AOM) is shown as a grey-scaled background. **a** ii Fast-polarisation dynamics SRS images of lipid order in a MLV taken at different times of the observation sequence shown; the AOM is shown as a grey-scaled background. Zoomed regions at the upper part of the MLV contour show no change in lipid order during the measurement over tens of seconds. **a** iii Fast-polarisation dynamics of lipid order in a thin lipid membrane. Coloured sticks show *S*_2_ and *φ*_2_ and the grey-scale background shows the AOM amplitude. **b** i SRS flow cytometry (SRS-FC) setup. **b** ii Colour-coded constrained principle component analysis (CPCA) scatter plot of SRS-FC spectra from mixed PMMA (red), PS (blue) and PCL (green) beads. The principle components (PC 1 and PC 2) are distinguished subpopulations of mixed polymer beads according to the distinct Raman spectra. Data were acquired in 6 s using a bead mixture with a concentration of 2% solids. The beads were 10 μm in diameter. **b** iii (left) Colour-coded CPCA scatter plot from SRS-FC analysis of lipid amount in 3T3-L1 cells with principle components differentiated through quantification of distinct chemical compositions inside single cells. Data were acquired in 3 s. **b** iii (right) SRS images of the two 3T3-L1 cell types. **c** i Illustration of the generation and amplification of a new orbital angular momentum (OAM) laser in a configuration with no initial OAM using a Hermite-Gaussian (TEM) laser. **c** ii, iii Simulation of the generation and amplification of a new OAM mode from initial configurations with no net OAM. **c** ii The initial seed TEM modes in *x*- and *y*-directions (top and bottom, respectively). **c** iii The new OAM mode electric field components at *z* = 3.5 mm in *x*- and *y*-directions (top and bottom, respectively). The new mode is linearly polarised in the *x*- and *y*-directions with *l*_1x_ = *l*_1y_ =  − 1 from an initial seed polarised in the *x*-direction with a TEM01 mode and in the *y*-direction with a TEM10 mode that is *π*/2 out of phase with respect to the TEM01 mode polarised in *x*. The pump is a Gaussian laser polarised at 45°. Projections in the (x,y) plane (blue-white-red) show the normalised vector potential (ϕ0) field envelope of the new OAM mode at the longitudinal slice where the laser intensity is maximum. The envelope of the 3D laser intensity is also shown in blue-green-red colours and normalised vector potential isosurfaces in blue and red. The values of the laser vector potential illustrated by the isosurfaces are shown in c (ii) and c (iii). **a** reproduced with permission from the OSA [259]. **b** reproduced with permission from the OSA [260]. **c** Adapted from [46] and licenced under CC BY 4.0.

Figure 6a.i shows a comparison of conventional polarisation SRS with that from Hofer’s fast-polarisation SRS on a multi-lamellar lipid vesicle (MLV). The fast-polarisation SRS image in the bottom of Fig. 6a i was obtained in 1 s which is two orders of magnitude faster than the conventional-SRS image (top) using the same incident powers, number of pixels and dwell time per pixel. Figure 6a ii shows sub-second frame-rate imaging of a MLV using double EOM-AOM modulation SRS at two instances in time. The measurement technique was remarked to have little effect on the lipid order properties during the measurement. Hofer et al.[259] were able to observe second-timescale dynamics in *thin* lipid membranes down to the cell plasma membrane using fast-polarisation-resolved SRS as shown in Fig. 6a iii.

Flow cytometry (FC) is one of the most important technologies for high-throughput single-cell analysis. FC is a technique used to measure physical/chemical characteristics of a population of cells or particles suspended in a fluid [59, 261]. The fluid suspension flows through the instrument detectors for fluorescent labelling which is the primary approach for cellular analysis in FC. Figure 6b i shows an optical FC setup [260]. However, for small molecules, the fluorescent tags can perturb the biological function of the species. In addition, non-specific binding of fluorescent labels as well as cellular autofluorescence can also reduce the clarity of the result. SRS flow cytometry (SRS-FC) non-invasively detects chemical cell content but conventional techniques suffer slow acquisition rates.

Zhang et al. [260] have recently demonstrated label-free high-throughput single-particle SRS-FC with a 32-channel multiplexing technique. Their technique measured single-particle chemistry at a rate of 5 μs per SRS spectrum, approaching that of standard fluorescence-based FC. The SRS-FC technique was based on broadband laser excitation and a multiplex spectral detection system. The systems allowed the acquisition of 200,000 spectra per second, more than 11,000 particles per second. The subpopulations of species, such as mixed polymer beads and 3T3-L1 cells, could be separated and distinguished through compositional principle component analysis (CPCA) of the SRS signals. The principle components were designated according to their Raman spectra. An agglomerative clustering procedure was performed on the resulting CPCA spectral matrix. This procedure assumed the number of cluster groups (*κ*) was known to separate the clusters of principle components in the CPCA analysis. Figure 6b ii shows the CPCA of the SRS spectra for a mixture of three types of beads: poly-methyl-methacrylate (PMMA), polystyrene (PS) and polycaprolactone (PCL), all with a 10-μm mean diameter, mixed at a 2:1:1 ratio of PMMA:PS:PCL and a final concentration of 2% solids in the fluid. The flow speed was ≈ 0.16 ms^−1^, the SRS-FC data was acquired in 6 s. The CPCA plot (Fig. 6b ii) shows three distinct clusters of principle components. The agglomerative clustering procedure (*κ* = 3) allowed the quantification of ~ 7100 PMMA bead (red), ~ 3400 PS beads (blue) and ~ 3600 PCL beads (green) as shown in Fig. 6b ii. Their measurement demonstrated the ratio of ≈ 2:1:1 (PMMA:PS:PCL) at a throughput rate of ~ 2350 particles per second and that their multiplex SRS-FC system, paired with the CPCA analysis, could distinguish different chemical components with small spectral differences. Zhang et al. [260] were able to detect beads as small as 1 μm and were even able to detect single *Staphylococcus aureus* bacteria flowing through the laser focus highlighting the potential to characterise subcellular organelles with SRS-FC.

Zhang et al. [260] also demonstrated the discrimination of 3T3-L1 cells at different stages of cell differentiation according to their difference in lipid amount using SRS-FC. After insulin-induced differentiation, 3T3-L1 cells acquire an adipocyte-like phenotype with a significantly increased amount of triglycerides which aggregate to form large lipid droplets. This aggregation of triglycerides causes the intensity of the methylene symmetric vibration at $\Delta \overset{\sim }{\nu }$ = 2850 cm^−1^ from fatty-acid acyl chains to become stronger compared to that of non-differentiated cells which provides the means for CPCA analysis. Figure 6b iii shows the CPCA scatter plot of the cell mixture measured by Zhang et al. [260] which were separated using the agglomerative clustering approach. The insert SRS images (Fig. 6b iii, right) show a non-differentiated 3T3-L1 cell and a differentiated cell with the formation of large lipid droplets.

Twisted Laguerre-Gaussian lasers, with orbital angular momentum (OAM) and characterised by doughnut-shaped intensity profiles, are of great interest to a number of growing research fields such as ultra-cold atoms [262–264], microscopy and imaging [265, 266], atomic and nanoparticle manipulation [267, 268], ultra-fast optical communication [269, 270], quantum computing [271], astrophysics [272] and plasma accelerators [47]. Spiral phase plates or computer-generated holograms are usually used to generate visible light with OAM [273–276]. Spiral phase plates can produce light with predefined OAM modes. By using plasma as an optical medium to generate and amplify laser pulses with OAM and relativistic intensities, well above the damage threshold of optical devices, could provide for high-energy-density science and applications. Plasmas also allow for greater flexibility in the level of OAM in the output laser beam than conventional optics. Vieira et al. [46] have shown that SRS in nonlinear optical media with Kerr nonlinearity (e.g. plasmas, optical fibres and nonlinear optical crystals) can be used to generate and amplify OAM light. The authors show that it is possible to generate and amplify light with OAM when no net OAM is initially present. Figure 6a i illustrates the process in which the pump EM fields can have different OAM components in both transverse directions *x* and *y* (blue and orange in Fig. 6a i). *l*_0x_ is the pump electric field component of OAM in the *x* direction. Likewise, *l*_0y_ is the pump electric field component of OAM in the *y* direction. The initial seed electric field component has an OAM component *l*_1x_. After interacting with the plasma, the pump is depleted, and a new electric field component appears in the seed with OAM *l*_1y_ = *l*_1x_ + *l*_0x_ − *l*_0y_.

The authors [46] use an analytical theory for arbitrary transverse laser field envelope profiles and particle-in-cell simulations for plasma. Stimulated Raman backscattering in plasma is a three-wave mode coupling process in which a pump pulse decays into an electrostatic (Langmuir) plasma wave as well as a counter-propagating seed laser. The plasma can be viewed as a high-intensity mode converter. The presence of OAM in the pump and/or seed results in additional matching conditions that ensure the conservation of angular momentum of the pump when the pump decays into a scattered electro-magnetic wave and a Langmuir wave [40].

Particular superpositions of Hermite-Gaussian modes TEM modes are mathematically equivalent to Laguerre-Gaussian modes [277]. Vieira et al. [46] therefore explore the use of Stimulated Raman backscattering to generate and amplify light with OAM using TEM laser beams with no initial net OAM. Each Hermite-Gaussian beam in the simulation is described by TEM_*m*,*n*_, where *m* and *n* represent the Hermite-Gaussian mode index. Figure 6c ii and iii show the 3D simulation results from the setup shown in Fig. 6c i. The simulations show that SRS results in a new OAM mode with *l*_1_ = 1 linearly polarised at 45°. The field topology of the seed normalised vector potential changes from plane isosurfaces in Fig. 6c ii, to helical isosurfaces in (Fig. 6c iii). Hence, light with OAM has been generated from light with no net OAM. The authors [46] note that their results could be extended to other nonlinear optical media with Kerr nonlinearity. In the case of plasma, the interaction between the seed light and the pump light occurs via an electron Langmuir wave. This interaction ensures that the frequency, wavenumber and OAM matching conditions are conserved.

### Coherent Anti-Stokes Raman Scattering Microscopy

**Fig. 7:**
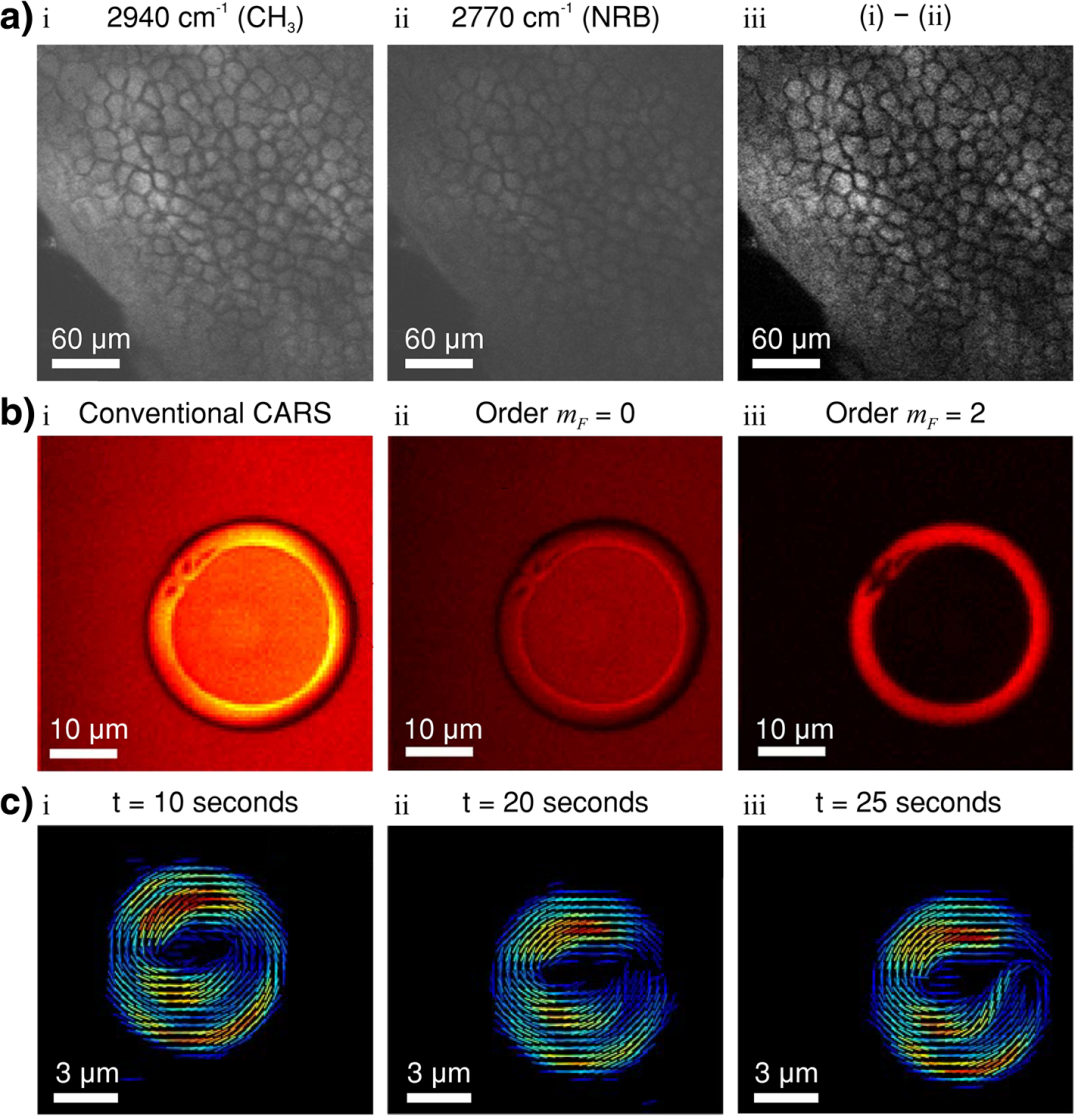
**a** Simultaneous two-colour CARS imaging with real-time nonresonant background subtraction from a mouse tissue sample at a surface depth of 45 μm. **a** i CARS image acquired at 2940 cm^−1^ (CH3 stretching vibration). **a** ii Off-resonance background CARS image at 2770 cm^−1^. **a** iii Background-free image of (i) at 2940 cm-1 after subtraction of the nonresonant background (ii). **b** Multi-lamellar lipid vesicle (MLV) imaged with conventional (i) and symmetry-resolved CARS (SR-CARS) at 1133 cm-1 (ii) and (iii). ii The incident circularly polarised pump, Stokes, probe and anti-Stokes light have co-rotating handedness (*m*_F_ = 0). iii the incident circularly polarised pump, probe and anti-Stokes light have co-rotating handedness and the Stokes light has counter rotating handedness (*m*_F_ = 2). **c** High-speed polarisation-resolved CARS image sequence on a MLV moving over the sample surface taken at different times of the observation sequence shown as a composite image of *S*_2_ and *φ*_2_ as coloured sticks and with the acousto-optic modulation (AOM) as a grey background. **a** reproduced with permission from the OSA [278]. **b** Adapted from [289] and licenced under CC BY 4.0. **c** reproduced with permission from the OSA [259].

CARS results from an induced anti-Stokes scattering of radiation, *ω*_AS_, which is enhanced when *ω*_p_ − *ω*_S_ = *ω*_OSC_. One of the main challenges with CARS microscopy is the nonresonant background. The existence of the nonresonant background can either distort or even saturate the resonant signal of Raman peaks, which reduces the image contrast. Qin et al. [278] have recently demonstrated multi-colour background-free coherent anti-Stokes Raman scattering microscopy using an all-fibre, low-cost, multi-wavelength *time lens* source. A time lens, in analogy to a spatial lens, is simply a quadratic optical phase modulator in time, which can be approximated by a portion of a sinusoidal phase modulator [279–281]. Three different wavelength picosecond pulse trains were provided by the time lens source, at 1064.3 nm (stable), 1052–1055 nm (tuneable) and 1040–1050 nm (tuneable). The time lens was used to apply temporal quadratic phase modulation to a continuous-wave laser to broaden its spectral bandwidth [279, 282–288]. In this instance, the time lens was applied with fibre-integrated electro-optic radio-frequency phase modulators. The phase modulation and pulse synchronisation were derived from a mode-locked Ti:Sapphire laser that provided synchronised multi-colour picosecond pulses with dispersion compensation. Electronic tuning of the pulse delay was used to achieve temporal overlap between the pump and Stokes laser pulse trains, which is a convenient substitution for mechanical optical delay paths. Two of the three wavelengths of light from the time lens source were used for two-colour on-resonance imaging and the third wavelength for off-resonance (nonresonant background subtraction) imaging. Pixel-to-pixel wavelength switching was achieved, which provided simultaneous two-colour CARS imaging with real-time nonresonant background subtraction. Qin et al. [278] demonstrated the technique with an excised fresh tissue sample from a mouse ear and imaged molecular stretching vibrations at 2845 cm^−1^ (CH_2_) and 2940 cm^−1^ (CH_3_) and non-resonance background at $\Delta \overset{\sim }{\nu }$ = 2770 cm^−1^. Figure 7a i–iii shows the process applied to the Raman peak of CH_3_ stretching vibration from the mouse ear tissue sample.

The nonresonant background signal in CARS can also be suppressed by applying an external static electric field to the sample known as electro-CARS. Capitaine et al. [120] demonstrate this electro-optical technique on n-alkanes in solution with broadband multiplex coherent anti-Stokes Raman scattering spectroscopy. The nonresonant background is suppressed due to the orientation response of the molecules to the electric field. The molecular orientation is related to the induced electric dipole moment. The enhancement of the CARS signal-to-noise ratio was achieved in the case of the CH_2_ and CH_3_ symmetric/asymmetric stretching vibrational modes.

Conventional CARS provides information about the chemical nature but not about the molecular organisation or symmetry in the system. The Cartesian components of the nonlinear susceptibility tensor *χ*^(3)^ represent the vibrational symmetry properties of the material [290, 291]. These tensor elements can be extracted with polarisation-resolved coherent Raman scattering schemes [72, 74, 169, 259, 292–295]. However, these schemes often involve the acquisition of multiple images from different polarisation angles requiring long acquisition times due to limits imposed by polarisation tuning [259] and time-consuming post-processing [67].

Cleff et al. [289] have recently demonstrated a label-free microscopy technique that uses circularly polarised light to probe the symmetry as well as the chemical fingerprint of the probed sample in a single acquisition. This symmetry-resolved CARS (SR-CARS) depends on both the presence of (ro-)vibrational modes as well as their local organisation. By switching between combinations of left- and right-handed circular polarisation states for the involved fields, the individual symmetry contributions of the sample can be imaged. This technique offers a straightforward means to access the local organisation of (ro-)vibrational bonds with improved image contrasts (with 1 to 2 orders of magnitude) for anisotropic samples, as well as improved chemical selectivity without post-processing and independently of sample orientation in the transverse plane. In addition, SR-CARS provides higher chemical selectivity with the contrast in symmetry characteristics, which are not accessible with conventional spontaneous Raman or SRS microscopy.

Multi-lamellar lipid vesicles (MLVs) are made of a tight packing of lipid layers forming a ring of highly ordered matter with twofold symmetry and a lipid orientation distribution close to a Gaussian angular shape [74].

Figure 7b i shows a conventional CARS image of an aqueous MLV at $\Delta \overset{\sim }{\nu }$ = 1133 cm^−1^ (C-C stretching vibration) which illustrates the expected poor contrast due to the nonresonant background [289]. Figure 7b ii and iii show the zeroth and second-order ${m}_{\overline{F}}$-value image of the same MLV as in Fig. 7b ii. The ${m}_{\overline{F}}$-value is the summation of the light circular polarisation handedness quantum numbers of the incident light beams:

15 $$ {m}_{\overline{F}}={m}_{\mathrm{p}}-{m}_{\mathrm{s}}+{m}_{\mathrm{p}\mathrm{r}}-{m}_{\mathrm{as}} $$

When light with field tensor $\overline{F}$ probes matter with nonlinear susceptibility tensor *χ*^(3)^, in a CARS process, the light probes only the parts of the matter with identical rotational invariant symmetries (i.e*.* identical ${m}_{\overline{F}}$). Hence, by engineering the field tensor of the light, specific sample symmetries can be directly read out, creating a symmetry-based image contrast mechanism [289]. The aqueous solution surrounding the MLV is only visible in the ${m}_{\overline{F}}=0$ image (Fig. 7b ii) due to its purely isotropic nature. Background-free imaging of the MLV with superior contrast with respect to conventional CARS is shown in Fig. 7b iii at ${m}_{\overline{F}}=2$, which results from the symmetric microscopic organisation of the lipids in the MLV. Imaging at ${m}_{\overline{F}}=4$ (not shown) lacked sufficient signal strength to provide an image of the MLV due to the lack of anti-symmetry in the lipid organisation.

As with SRS, the CARS signal is sensitive to the polarisation of the incident light when the orientation of the scattering species is ordered. Polarisation-resolved CARS (PR-CARS) requires monitoring of the CARS signal response depending on the relative rotation of the incident light polarisations (pump and Stokes) to the sample, in species with ordered orientations. Provided that the molecular bonds are oriented, the detected intensity of the anti-Stokes signal is maximised when the incident polarisations lie along the averaged direction of the bonds. The ability to monitor lipid order without the need for fluorescent labels can provide information on lipid packing properties. As mentioned, PR-CARS schemes often involve long acquisition times due to limits imposed by polarisation tuning and time-consuming post-processing.

In addition to fast-polar-SRS, Hofer et al. [259] have recently demonstrated fast-polar-CARS imaging with combined electro-optic polarisation and acousto-optic amplitude modulations. Figure 7c shows fast-polarisation CARS with similar sensitivity to that of SRS (shown in Fig. 6a). Despite the requirement of lock-in amplification for the detection of low modulation over a large nonresonant background, the fast-polarisation technique demonstrated by Hofer et al. [259] can considerably improve the signal-to-noise ratio in CARS imaging. Despite the robustness of MLVs, occasional alteration of molecular order in MLVs could be observed at the time scale accessible in Hofer’s experiment (0.25–1 s per image). MLVs could detach from the sample surface, inducing motion (Fig. 7c) or shape change. The modifications observed in Fig. 7c were attributed to a local membrane disruption, followed by its spontaneous reformation. Hofer et al. [259] demonstrated the possibility of visualising local modification during MLV displacement that was not accessible using the minute-time-scale conventional polarisation Raman experiments.

There have also been a number of developments in CARS flow cytometry (CARS-FC) [58, 296, 297]. However, these techniques were shown to be much slower than fluorescence-based FC. Out-of-focus microparticles can randomly impede CARS-FC and the fluid often generates a strong nonresonant background limiting CARS-FC from achieving high-throughput single-cell analysis. Recently, however, O’Dwyer et al. have demonstrated that it is possible to significantly enhance the fraction of unambiguously and instantly recognised in-focus microparticles, in unconstrained flows by co-monitoring CARS-FC with linear scattering of light.

CARS is invariably performed with two synchronised picosecond laser sources owing to the coherence life time of Raman resonance. Ti:Sapphire oscillators [168] or optical parametric oscillators pumped by a picosecond frequency-doubled Nd:Vanadate laser [53] are the instruments of choice, which are generally very expensive and the synchronisation mechanisms can be challenging. In addition, the spectral drift in the pump wavelength can introduce errors in the calculation of *ω*_osc_. Langbein et al. [124] have demonstrated CARS micro-spectroscopy using a single Ti:Sapphire laser oscillator and simple passive optical elements. Vibrational excitation, tuneable over a large spectral range with adjustable spectral resolution, was achieved by spectral selection with dichroic mirrors and linear chirping by glass elements.

### Tip-Enhanced Dual Wavelength Coherent Anti-Stokes Raman Scattering Microscopy

TERS offers spatial resolution far beyond the diffraction limit of the probing light. The more conventional technique is to directly illuminate the tip-sample cavity [86]. This technique achieves the desired resolution (beyond the diffraction limit) by forcing the evanescent light into the far field image formation. However, the far field light presence in the tip-sample cavity generates an unfavourable background light source. It is possible to perform TERS by coupling the far-field excitation light to the tip a few tens of micrometres from the tip apex [298]. Femtosecond laser pulses can be coupled to the tip surface by shining the light on a grating fabricated on the tip surface. The SPPs then propagate to the tip apex and generate background-free localised optical excitation [210].

**Fig. 8:**
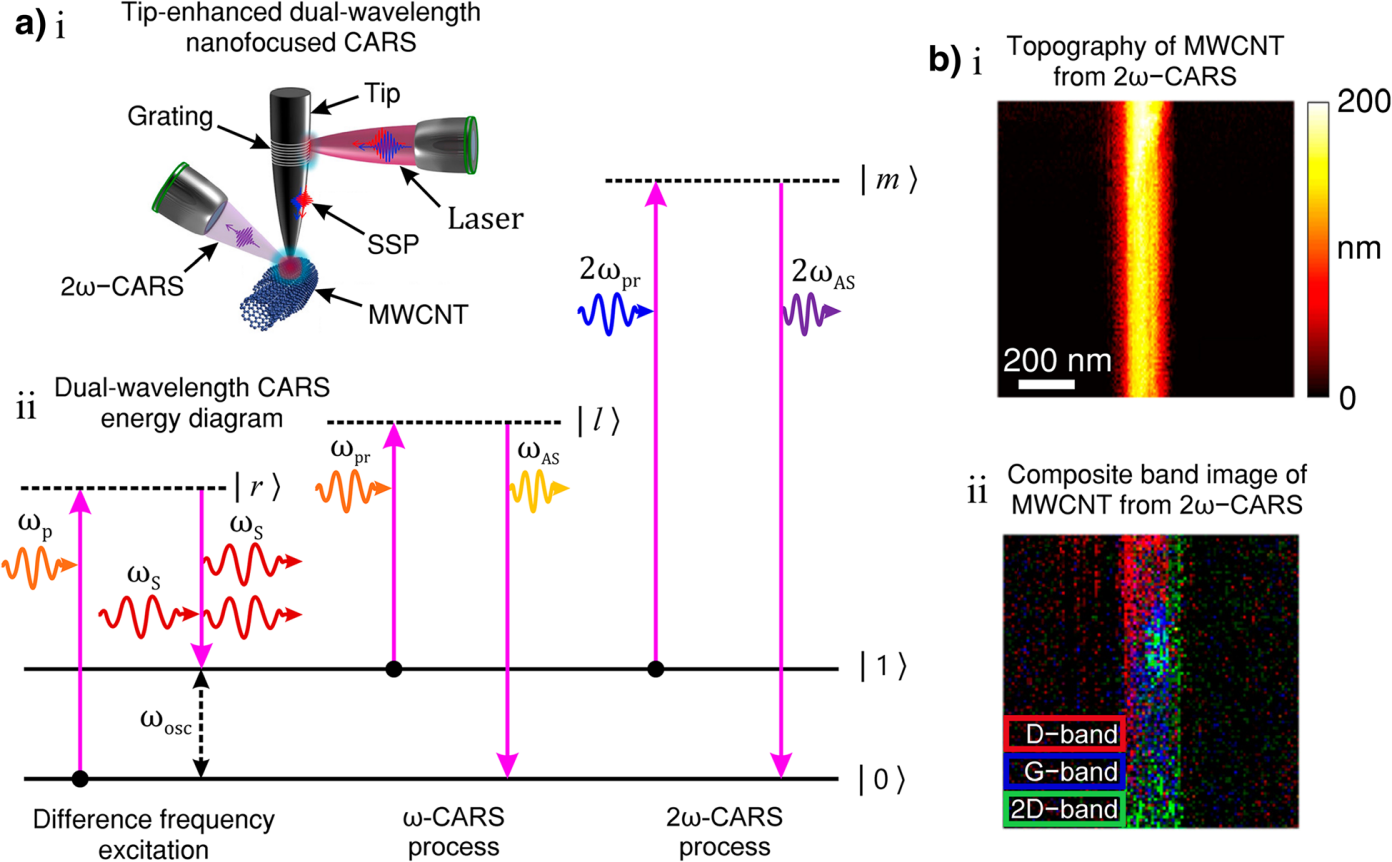
**a** i Illustration of tip-enhanced dual-wavelength nanofocused CARS on a multi-walled carbon nanotube (MWCNT). **a** ii Energy diagram for ω-CARS and 2ω-CARS. When the difference frequency between pump and Stokes light matches the vibrational mode of the molecule, it is resonantly excited. When ω and 2ω-probe light is simultaneously incident within the dephasing time, ω- and 2ω-CARS photons are respectively generated. **b** i Simultaneous topographical 2ω-CARS imaging of a MWCNT. **b** ii Composite image of three 2ω-CARS images of the MWCNT using the 2ω-CARS spectrum from D- (red), G- (blue) and 2D- (green) bands. Reprinted with permission from [155]

Toma et al. [298] previously demonstrated selective excitation of a single Raman mode and its CARS imaging of CNT using ultra-fast SPP pulse nanofocusing using an Au tapered tip. In a more recent publication, seminal work by Tomita et al. [155] demonstrated simultaneous nanofocusing of ultra-fast SPP pulses at 440 and 800 nm, which were coupled with a common diffraction grating structure. Figure 8a i, illustrates the scheme. The Al-tapered tip had an apex radius of ≈ 35 nm. Selective CARS microscopy that combined an 800 nm (*ω*) SPP pump pulse and a 440-nm (2*ω*) SPP probe pulse was achieved. Figure 8a ii illustrates the energy level process of *ω*- and 2*ω*-CARS. The pump pulse achieves selective vibrational excitation by spectral focusing [299–302]. Raman shift intensities with this 2ω-CARS scheme were reported to increase by as much as 4 compared with that of ω-CARS for monolayer graphene. The selectivity of vibration band excitation and background noise suppression were confirmed on the CARS intensity probed by a 2*ω*-SPP plasmon pulse for a monolayer graphene sample. Venezuela et al. [8] reported the Raman lines in graphene associated with both phonon-defect processes (such as the D line at $\Delta \overset{\sim }{\nu }$ ~ 1350 cm^−1^) and two-photon processes (such as the 2D line). The 2D-band intensity in graphene was reduced monotonously when the defect concentration was increased, contrary to the D-band. Tomita et al. [155] applied their multi-vibrational-mode 2ω-CARS imaging method to a multi-walled CNT (MWCNT) at the D, G and 2D bands. This dual-wavelength nanofocusing technique could open new nanoscale micro-spectroscopy and optical excitation schemes in SPM, such as sum frequency mixing, two-photon excitation (*ω*+2*ω*) and pump-probe schemes.

Figure 8b i shows a topography image of a MWCNT with a diameter of ~ 175 nm measured by the Al-tapered tip. Figure 8b ii shows a composite image of three 2ω-CARS images of the MWCNT using the 2ω-CARS spectrum from D- (red), G- (blue), and 2D- (green) bands. In ref. [155], the D- and 2D-band showed a negative correlation (in agreement with ref. [8]) except for the central part of the MWCNT. The 2D- and G-bands were intense near the central part of the MWCNT. Tomita et al. [155] indirectly estimated the spatial resolution of their technique to be less than 90 nm by taking the profile of the 2D-band signal across the axis of the MWCNT.

## Conclusions

This review detailed the numerous applications of Raman spectroscopy and its advanced derivatives: stimulated Raman scattering, coherent anti-Stokes Raman scattering and surface- and tip-enhanced Raman spectroscopy. A description of the fundamental physics that underpins these techniques has been provided. Experimental considerations have been discussed with examples of typical instrumentation used. Examples of the analysis techniques employed to interpret the Raman spectroscopic data were presented and discussed.

The Raman effect now underpins prominent spectroscopic techniques in biology, medicine, crystallography and flow cytometry and has gained interest in plasma physics. It is employed as a non-invasive label-free chemically selective hyperspectral imaging technique with recent advances enabling the probing of molecular orientation and chemical composition. SRS and CARS are used to enrich signal detection at specified wavelengths associated with vibrational modes that are prescribed for spectral-selective imaging. Unlike SRS, CARS carries with it a nonresonant background contribution to the spectrum. This review detailed some of the efforts to suppress this unfavourable contribution.

Surface-enhanced Raman scattering is an ultrasensitive Raman technique that has enabled the detection of trace amounts of molecular species in samples that would otherwise be undetectable in spontaneous- or coherent Raman scattering techniques. The enhancement effect is largely associated with the plasmonic activity of the sample surface, which augments the light-matter interaction. This enhancement effect is optimised by tuning the plasmons associated wavelength with plasmonically active surface nanostructures.

Tip-enhanced Raman scattering spectroscopy is a relatively new technique that can capture hyperspectral images with spatial resolution beyond the diffraction limit of light. As light is fundamental to the Raman effect, the spatial resolution offered by TERS is so far unparalleled by other Raman scattering techniques. The surface plasmon wavelength can also be tuned for TERS techniques and recent advances have exploited surface plasmon polaritons to focus evanescent light at the tip apex with light coupled remotely from the tip apex. This technique has yielded enhanced the signal-to-noise ratio by removing the far-field light from the apex region. Recent advances have demonstrated this technique with dual-wavelength CARS.

## Data Availability

Not applicable

## Abbreviations

AFM: Atomic force microscopy
AOM: Acousto-optic modulator
AS: Ammonium sulphate
CARS: Coherent anti-Stokes Raman scattering
CARS-FC: CARS flow cytometry
CNT: Carbon nanotube
CPCA: Compositional principle component analysis
CRS: Coherent Raman scattering
DBR: Distributed Bragg reflection
DFB: Distributed feedback
E-CARS: Epi-scattered CARS
EM: Electro-magnetic
FC: Flow cytometry
F-CARS: Forward scattered CARS
HEMTs: High electron mobility transistors
LSPR: Local surface plasmon resonance
MLV: Multi-lamellar lipid vesicle
MoS_2_: Molybdenum disulphide
MWCNT: Multi-walled carbon nanotube
NA: Numerical aperture
OAM: Orbital angular momentum
PCL: Polycaprolactone
PMMA: Poly-methyl-methacrylate
PR-CARS: Polarisation-resolved CARS
PS: Polystyrene
SERS: Surface-enhanced Raman scattering
SFM: Shear force microscopy
SLM: Single-longitudinal mode
SPM: Scanning probe microscopy
SPP: Surface plasmon polariton
SR-CARS: Symmetry-resolved CARS
SRS: Stimulated Raman scattering
STM: Scanning tunneling microscopy
TEM: Transverse electro-magnetic mode
TERS: Tip-enhanced Raman scattering
VBG: Volume Bragg-grating
